# Effect of nutrition-sensitive agriculture interventions with participatory videos and women's group meetings on maternal and child nutritional outcomes in rural Odisha, India (UPAVAN trial): a four-arm, observer-blind, cluster-randomised controlled trial

**DOI:** 10.1016/S2542-5196(21)00001-2

**Published:** 2021-03-31

**Authors:** Suneetha Kadiyala, Helen Harris-Fry, Ronali Pradhan, Satyanarayan Mohanty, Shibanath Padhan, Suchitra Rath, Philip James, Emily Fivian, Peggy Koniz-Booher, Nirmala Nair, Hassan Haghparast-Bidgoli, Naba Kishor Mishra, Shibanand Rath, Emma Beaumont, Heather Danton, Sneha Krishnan, Manoj Parida, Meghan O'Hearn, Abhinav Kumar, Avinash Upadhyay, Prasanta Tripathy, Jolene Skordis, Joanna Sturgess, Diana Elbourne, Audrey Prost, Elizabeth Allen

**Affiliations:** a Department of Population Health, London School of Hygiene & Tropical Medicine, London, UK; b Department of Medical Statistics, London School of Hygiene & Tropical Medicine, London, UK; c Digital Green, New Delhi, India; d DCOR Consulting, Bhubaneshwar, India; e Voluntary Association for Rural Reconstruction and Appropriate Technology, Kendrapara, India; f Ekjut, Chakradharpur, India; g JSI Research & Training Institute, Arlington, VA, USA; h Institute for Global Health, University College London, London, UK; i Environment, Technology and Community Health Consultancy Service, Mumbai, India; j Jindal School of Environment and Sustainability, Jindal Global University, Haryana, India; k Friedman School of Nutrition Science and Policy, Tufts University, Boston, MA, USA

## Abstract

**Background:**

Almost a quarter of the world's undernourished people live in India. We tested the effects of three nutrition-sensitive agriculture (NSA) interventions on maternal and child nutrition in India.

**Methods:**

We did a parallel, four-arm, observer-blind, cluster-randomised trial in Keonjhar district, Odisha, India. A cluster was one or more villages with a combined minimum population of 800 residents. The clusters were allocated 1:1:1:1 to a control group or an intervention group of fortnightly women's groups meetings and household visits over 32 months using: NSA videos (AGRI group); NSA and nutrition-specific videos (AGRI-NUT group); or NSA videos and a nutrition-specific participatory learning and action (PLA) cycle meetings and videos (AGRI-NUT+PLA group). Primary outcomes were the proportion of children aged 6–23 months consuming at least four of seven food groups the previous day and mean maternal body-mass index (BMI). Secondary outcomes were proportion of mothers consuming at least five of ten food groups and child wasting (proportion of children with weight-for-height Z score SD <–2). Outcomes were assessed in children and mothers through cross-sectional surveys at baseline and at endline, 36 months later. Analyses were by intention to treat. Participants and intervention facilitators were not blinded to allocation; the research team were. This trial is registered at ISRCTN, ISRCTN65922679.

**Findings:**

148 of 162 clusters assessed for eligibility were enrolled and randomly allocated to trial groups (37 clusters per group). Baseline surveys took place from Nov 24, 2016, to Jan 24, 2017; clusters were randomised from December, 2016, to January, 2017; and interventions were implemented from March 20, 2017, to Oct 31, 2019, and endline surveys done from Nov 19, 2019, to Jan 12, 2020, in an average of 32 households per cluster. All clusters were included in the analyses. There was an increase in the proportion of children consuming at least four of seven food groups in the AGRI-NUT (adjusted relative risk [RR] 1·19, 95% CI 1·03 to 1·37, p=0·02) and AGRI-NUT+PLA (1·27, 1·11 to 1·46, p=0·001) groups, but not AGRI (1·06, 0·91 to 1·23, p=0·44), compared with the control group. We found no effects on mean maternal BMI (adjusted mean differences *vs* control, AGRI −0·05, −0·34 to 0·24; AGRI-NUT 0·04, −0·26 to 0·33; AGRI-NUT+PLA −0·03, −0·3 to 0·23). An increase in the proportion of mothers consuming at least five of ten food groups was seen in the AGRI (adjusted RR 1·21, 1·01 to 1·45) and AGRI-NUT+PLA (1·30, 1·10 to 1·53) groups compared with the control group, but not in AGRI-NUT (1·16, 0·98 to 1·38). We found no effects on child wasting (adjusted RR *vs* control, AGRI 0·95, 0·73 to 1·24; AGRI-NUT 0·96, 0·72 to 1·29; AGRI-NUT+PLA 0·96, 0·73 to 1·26).

**Interpretation:**

Women's groups using combinations of NSA videos, nutrition-specific videos, and PLA cycle meetings improved maternal and child diet quality in rural Odisha, India. These components have been implemented separately in several low-income settings; effects could be increased by scaling up together.

**Funding:**

Bill & Melinda Gates Foundation, UK AID from the UK Government, and US Agency for International Development.

## Introduction

Undernutrition among women and children causes adverse pregnancy outcomes, impairs children's physical and cognitive development, and increases morbidity and mortality risk. Around 51 million children (aged <5 years) are wasted (weight-for-height Z score SD <–2) and 154 million women aged 15–49 years are underweight (body-mass index [BMI] <18·5 kg/m^2^).^1^ Scaling up evidence-based nutrition-specific interventions to address the immediate determinants of undernutrition is necessary but insufficient to eliminate undernutrition,^2^ and the second Sustainable Development Goal (SDG) of zero hunger is not achievable at the current pace. Recognising the importance of other sectors, the SDG 2.3 target aims to double agricultural productivity and incomes of subsistence farmers, particularly women.

**Research in context**

**Evidence before this study**

When we initially designed the study, an influential 2013 review by Ruel and colleagues called for more rigour in the design and impact evaluations of nutrition-sensitive agriculture (NSA) interventions. In 2018, the authors updated this review to include studies published between January, 2014, and January, 2017. They found that some NSA interventions improved child diet quality, but evidence was sparse on child wasting, maternal nutrition, and cost-effectiveness, and too few studies were from south Asia. Further, the authors called for an expansion of behaviour change communication approaches beyond traditional nutrition education, to build skills and capacities of household members in meal planning, budgeting, hygiene, and health service use.

We updated this 2018 review using the same search strategy, identifying studies published between Jan 1, 2017, and April 1, 2020, in Scopus, PubMed, and Web of Science. We restricted our search to completed randomised controlled trials (RCTs) and excluded studies that solely tested food fortification in the absence of additional NSA activities. Our search yielded 350 records, of which nine articles from eight RCTs were eligible for inclusion (search terms, inclusion criteria, and results are in the appendix [pp 7–14]), giving a total of 17 articles from 12 NSA trials when combined with the 2018 review by Ruel and colleagues.

All NSA interventions provided some form of agriculture training and nutrition-specific behaviour change communication aiming to improve maternal knowledge and skills, but there was a paucity of evaluations of participatory and demand-driven approaches and none used participatory videos for peer-to-peer learning, peer support, building women's confidence, problem solving, and collective action. Effects of NSA interventions on children's minimum dietary diversity are mixed (four of eight trials found a positive effect), whereas effects on maternal dietary diversity are rarely and variously measured (two trials) and have shown null effects. All NSA trials have shown null effects on mean maternal body-mass index (tested in three trials) and prevalence of wasting (tested in six trials). The only south Asian NSA trials were in Nepal. In summary, these reviews reveal a lack of evaluation of participatory methods in NSA interventions, an evidence gap for maternal outcomes, and no NSA RCTs from India.

**Added value of this study**

We set up an NSA extension service of participatory videos, delivered through women's groups, integrated nutrition-specific videos within this service, and incorporated a nutrition-sensitive participatory learning and action approach to enhance the participatory component. To our knowledge, our study is the first to test different combinations of these approaches. We show that combinations of these intervention components can improve dietary diversity of mothers and children. However, we find no effects on maternal or child anthropometric status.

**Implications of all the available evidence**

Our trial results support existing literature showing that NSA interventions can improve children's minimum dietary diversity, and is the first to show that NSA interventions can also improve maternal minimum dietary diversity. However, together with most other NSA trials until April, 2020, our trial did not show improvements in maternal or child anthropometry. Child wasting continues to be an intractable problem and innovation in the prevention of child wasting is an urgent priority. Several trials, including ours, have documented evidence of impressive secular improvements in health, diets, and maternal anthropometric outcomes, indicating that existing government and other agency programmes are also working. Future efforts could therefore consider integrating effective NSA interventions, such as those tested in our trial, within larger scale, convergent multisectoral programme designs to increase impact.

21% of children in India are wasted, and almost a quarter of women are underweight.^3^ Over a half of Indians depend on subsistence farming. New approaches for integrating nutrition objectives into agricultural programmes (ie, nutrition-sensitive agriculture [NSA]) and evidence of their effects are needed.^4^, ^5^ We developed and tested three NSA interventions drawing on three promising approaches.

The first approach is agricultural extension, which provides services to improve farmers' technical knowledge, livelihoods, and food security,^6^ and is central to agricultural policy in most low-income and middle-income countries. The coverage of agricultural extension services is uneven and inequitable,^7^ and impacts on agricultural productivity are mixed.^6^ Agricultural extension could be strengthened and adapted to improve maternal and child nutrition.

The second approach is the use of participatory videos to make agricultural and nutrition interventions relevant and demand-driven.^8^, ^9^ Participatory video-making typically involves an iterative process: community groups view and discuss videos developed based on local needs, try practices described in the videos, and give feedback to shape the content of subsequent videos.^10^ In India, participatory videos increased agricultural productivity by 21%,^11^ and are deemed a feasible, scalable method for improving nutrition,^12^ but high-quality impact evaluations are needed.

The third approach is women's groups using participatory learning and action (PLA). In a PLA meeting cycle, groups identify and prioritise health problems, identify feasible strategies, implement these strategies with help from the wider community, and informally evaluate the process. In south Asia, women's groups practising PLA have improved infant survival, dietary diversity, and maternal anthropometry,^13^, ^14^ but improvements in child anthropometry are limited to studies where PLA was coupled with food supplementation through fortified flour or crèches.^15^, ^16^

Following a feasibility study in 2013^12^ and a pilot trial in 2014,^17^ we developed three NSA interventions for testing in Keonjhar district of Odisha, India, drawing on the approaches we have described before. We set up a nutrition-sensitive participatory video-based agricultural extension service delivered through women's groups; integrated nutrition-specific videos; and incorporated PLA. We did a cluster-randomised controlled trial (RCT) to test the hypothesis that one or more of the three intervention packages would improve maternal and child diets and nutritional status, compared with the control.^18^ In Keonjhar district, 86% of the population of 1·8 million engage in agriculture and 57% belong to Scheduled Castes and Scheduled Tribes—historically disadvantaged groups.^19^ In 2015–16, 30% of women in Keonjhar were underweight and 19% of children (aged <5 years) were wasted. Less than 10% of children (aged 6–23 months) were fed a WHO-defined minimally acceptable diet.^3^

## Methods

### Study design and participants

We did a parallel group, observer-blind, four-arm cluster RCT in Keonjhar district, Odisha, India (appendix p 1). A cluster was a village and its surrounding hamlets. Smaller adjacent areas (population <800 residents overall) were combined to achieve the required sample size per cluster.

The interventions were delivered at the cluster level and all women in intervention clusters were eligible to participate. We evaluated effects on trial participants: one child aged 0–23 months per household, their mothers or female primary caregivers when the mother was absent (hereafter mothers) aged 15–49 years, and the mother's spouse (or household head, if unavailable). Mothers with any disability impairing participation in the surveys, children with any disability affecting weight, standing height, or recumbent length, and household members who were not residents of the household for at least half a year before data collection were not eligible trial participants.

We obtained informed consent from village leaders for the participation of villages in the trial before randomisation and all data collection. Data collectors sought informed consent from adult participants in writing or by thumbprint, and, for children, from their primary caregivers.

Ethics approval was granted from the Odisha Government's Institutional Review Board, Research and Ethics Committee, Department of Health and Family Welfare, Government of Odisha (date approved Sept 3, 2016, letter number 141/SHRMU). Ethics approval was granted from the London School of Hygiene & Tropical Medicine (LSHTM) Interventions Research Ethics Committee (date approved Oct 10, 2016, reference number 11 357).

### Randomisation and masking

Clusters were randomly allocated to a control group or one of three intervention groups: NSA videos (AGRI group); NSA and nutrition-specific videos (AGRI-NUT group); or NSA videos and a nutrition-specific PLA cycle (AGRI-NUT+PLA group; appendix p 2).^18^ Staff from the LSHTM Clinical Trials Unit (JSt) did the randomisation remotely in two batches using Stata (version 16) and shared the password protected allocation file with SP. Stratified block randomisation allocated clusters in four administrative blocks in the ratio 1:1:1:1 (Ghatgaon, Harichandanpur, Patna, and Keonjhar sadar). Allocation was stratified by distance to nearest town (<10 km or ≥10 km) and low (<30%), medium (30–70%), and high (>70%) proportion of Scheduled Tribe or Scheduled Caste households, giving six strata. If more than one eligible child was present, all eligible children were listed in the survey tool, which was programmed to randomly select one.

Given the nature of the intervention, participants and intervention implementers were not masked to allocation. The research team, including the principal investigator and trial statistician, were masked to the allocation. Analyses were carried out by a statistician who was masked to allocation.

### Procedures

The participatory video approach used in all interventions was designed by Digital Green, an international non-governmental organisation. Community members initially identified appropriate topics and developed packages of practices—key actions to improve agricultural practices—to discuss in the videos. Then, other community members, often including government front-line workers, were filmed demonstrating and discussing these practices. Local facilitators then showed these videos to community groups, using low-cost, battery-operated projectors and pausing the video at specified points to facilitate discussion of the promoted practices. In doing so, groups discussed feasibility, interest, apprehension or experience, and barriers they might face, in adopting the practices. Facilitators visited group members at their homes or farms to ask whether they adopted the practices and could recall the messages. Finally, video viewership, knowledge recall, and adoption of practices were collated in a monitoring system, and qualitative feedback was gathered during facilitators' review meetings. Local implementers used this information to identify future video content.

We adapted this participatory video approach to make it nutrition sensitive and enhance participation, described in depth elsewhere.^18^, ^20^ Local, trained, salaried facilitators worked with self-help groups—an existing platform of women's groups involved in savings and lending activities (usually 20–25 members each).

The control group participants did not receive UPAVAN interventions. The AGRI group received an agricultural extension intervention with fortnightly women's groups that viewed and discussed participatory videos on NSA. Group participants who were pregnant or had a child (aged <2 years) received follow-up visits at home. NSA videos focused on practices related to at least one of four NSA pathways in the Theory of Change (appendix p 3): increasing availability of nutritious foods (eg, growing spinach and improved chicken keeping); increasing income (eg, System for Rice Intensification and reducing goat mortality); improving women's decision making in agriculture (eg, family budgeting and crop planning); and reducing workload for women who are pregnant and breastfeeding (eg, manually operated weeding machines). We also required all practices to do no harm (eg, handwashing after handling manure or chickens).

The AGRI-NUT group had fortnightly women's groups that viewed and discussed participatory NSA videos and nutrition-specific videos on maternal and child nutrition, with follow-up visits. On average, groups had one NSA and one nutrition-specific video per month. Nutrition-specific videos focused on age-appropriate child feeding practices, care during child illness, and maternal diets and rest.

The AGRI-NUT+PLA group had fortnightly women's group that viewed and discussed participatory NSA videos combined with PLA meetings, with follow-up visits. The PLA meeting cycle with women's groups comprised four phases. First, group members identified and prioritised nutrition problems. Second, they explored the causes and effects of prioritised problems, planned locally feasible strategies to address these, decided on roles and responsibilities for implementing the strategies, and shared their learning with the wider community. Third, groups implemented their strategies. Fourth, groups evaluated the process. Some PLA meetings in this group were discussion-based, whereas others were facilitated disseminations of videos on nutrition-specific topics. The videos in this group arose from the PLA meetings and were different from the nutrition-specific videos in AGRI-NUT. On average, groups had one NSA video and one PLA meeting (discussion-based or nutrition-specific video) per month (appendix p 4).^18^

Interventions, which lasted for 32 months, began with launch events to gain community support and invite women to participate, particularly to include younger women and self-help group strengthening activities. UPAVAN facilitators worked with women's groups to plan video dissemination schedules, invite women to meetings, disseminate and discuss videos in groups, conduct follow-up visits, monitor participation, and devise ways to improve coverage.

Government front-line nutrition and health workers (Anganwadi workers and Accredited Social Health Activists) in all groups, including the control, received a 2-day training course in maternal and child nutrition. In all groups, participants might have received services provided by the government or other non-governmental organisations.

Our Theory of Change articulates the inputs, activities, and hypothesised pathways to impact (appendix p 3). Each video addressed at least one pathway. We promoted practices based on their seasonal relevance, potential for uptake, and hypothesised impact on diets and nutritional status, which we determined using formative research,^21^ published evidence, local knowledge, and feedback. We used the transtheoretical model of behaviour change^22^ to reinforce and encourage adoption and maintenance of selected practices. We aimed to address women's individual-level barriers to adopting practices by increasing their knowledge, confidence, skills, and motivations, and addressed community-level barriers by strengthening group cohesion, collective problem-solving, community support, and diffusion of knowledge.

Digital Green coordinated implementation of all interventions. The Voluntary Association for Rural Reconstruction and Appropriate Technology, an Odisha-based non-governmental organisation, was responsible for implementation. John Snow Research and Training Institute led formative research and built technical capacity of partners. Ekjut, an Indian non-governmental organisation, provided technical assistance on PLA. The LSHTM led all research activities, with University College London's Institute for Global Health, and DCOR Consulting.

### Outcomes

The primary outcomes were percentage of children aged 6–23 months consuming at least four of seven food groups in the previous 24 h and the mean BMI of non-pregnant, non-postpartum (gave birth >42 days ago) mothers. Secondary outcomes were the percentage of mothers consuming at least five of ten food groups in the previous 24 h and the percentage of children with a weight-for-height Z score of less than −2 SD. Dietary diversity indicators were selected as validated measures of micronutrient adequacy^23^, ^24^ that could improve within the study timeframe.^5^ The cutoffs indicate 75% or more^25^ adequacy of intakes of multiple micronutrients for children and more than 60%^26^ for mothers. They were also selected because they indicate access to a diverse diet, which is important from a human rights perspective.

The choice of our anthropometric indicators, maternal BMI and child wasting, were based on interventions' impact pathways. BMI is a measure of chronic energy deficiency for adults and wasting indicates acute undernourishment for children. Both anthropometric indicators were amenable to change within the intervention timeframe, predict mortality and morbidity, and are globally used, which facilitates comparisons with other studies and global targets. Given the long and complex pathways to improved child linear growth, NSA interventions are unlikely to show impact on child stunting,^4^ and current consensus remains that NSA interventions should focus on improving diets rather than on reducing stunting.^5^

Other additional outcomes were mothers' and children's haemoglobin concentrations and mid-upper arm circumference (MUAC), and exploratory outcomes along the causal pathways included indicators of women's empowerment and agricultural production (appendix pp 15–16). The list of outcomes was published in the protocol.^18^

All outcomes were assessed through cross-sectional surveys at baseline and endline, 36 months later (at the end of the 32 month intervention). To reduce respondent burden, we randomly allocated half of the spouses to answer questions on empowerment, and the other half answered questions on household consumption.

Following Food and Agriculture Organization guidelines for women^23^ and WHO guidelines for children,^24^ dietary intakes were elicited using the free recall method and following prespecified probes. Dietary diversity assessments were standardised by observing each interviewer administer a 24 h recall and comparing results against a gold standard interviewer. Children's length and mother's height were measured using Seca 417 Infantometer (Seca, Germany) and 213 Stadiometers (Seca, Germany); their weight using MAX-CRUZER scales (Axis Electronics, India); MUAC using Médecins Sans Frontières produced standard MUAC tapes (MegaCare International, India); and haemoglobin using HemoCue Hb 301 machines (HemoCue, India). Certified laboratory technicians completed anthropometry standardisation on a set of ten mothers and ten children (aged 0–23 months) before data collection began. Technical error of measurement results for the laboratory technician teams at endline were less than 0·49 cm for height, 0·25 cm for adult MUAC, 0·06 kg for adult weight, 0·19 cm for child length, 0·12 cm for child MUAC, and 0·08 kg for child weight. Supervisors retrained interviewers and technicians with unsatisfactory results.

Data collection training took 5 weeks. A data quality assurance team did spot checks on 10% and back checks on 20% of households. All data at endline were captured on Android tablets using Open Data Kit software (version 1.29.3).

### Statistical analysis

We estimated that there would be on average 32 mother-child dyads with children aged 0–23 months and 24 mother-child dyads with children aged 6–23 months per cluster across 148 clusters, giving a total sample size of 4736 mother-child dyads. This sample size, with an intracluster correlation of 0·06 estimated from a previous study in the same district,^27^ had 80% power with a 5% level of significance to detect a 9% absolute difference in child minimum dietary diversity, between each intervention group separately and the control group, assuming a baseline of 22% of children with minimum dietary diversity.^28^ This sample size would also allow us to detect a difference in mean maternal BMI of 0·3 kg/m^2^ between each intervention group and the control group assuming an SD of 1·5 kg/m^2^, which equates to a standardised mean difference of 0·2. The detectable effects were determined by feasibility and our understanding that the expected effect sizes represent meaningful public health improvement. We did not adjust stringently for multiple comparisons, rather restricted testing to a set of prespecified outcomes and only compared each intervention group to the control group. We did not expect the differences between intervention groups to be as large as the difference between any one intervention group and control, so did not power the trial to detect these inter-intervention group differences because it would have led to an unfeasibly large trial.

The primary analysis of outcomes was by intention to treat and included all randomised clusters and participants. The analyses were cross-sectional, comparing outcomes in each of the intervention groups and the control group at endline. To adjust for the baseline measures of the outcomes, the analysis included all individuals at each timepoint linked by cluster. This approach uses the individual-level measures to effectively calculate cluster-level summaries of each outcome at baseline. The models therefore included a cluster-level summary of each outcome at baseline and the inclusion of a time variable additionally allowed us to estimate changes in outcomes over time in the study area. Each measure was analysed using separate generalised estimating equations (GEEs) to account for clustering. Statistical significance was taken at the 5% level (p<0·05). We carried out analyses adjusted only for baseline measures of the outcomes, and adjusted analyses that additionally included the stratification variables: distance to the nearest town and proportion of Scheduled Castes and Scheduled Tribes. The statistical analysis plan was approved by the trial steering committee, which included an independent statistician.

For the primary outcome of child minimum dietary diversity, we used a log-binomial GEE to estimate a relative risk (RR) for each intervention compared to control. For the primary outcome maternal BMI, we used a GEE with Gaussian link to estimate a mean difference in BMI between each intervention group and the control.

We restricted formal testing to a prespecified number of secondary outcomes, and appropriate GEEs were used to examine the effect of the interventions. For continuous outcomes, we report unadjusted and adjusted mean differences with 95% CIs. For binary outcomes, we report unadjusted and adjusted RR with 95% CI. Where there was evidence of non-normality in the continuous outcome measures, non-parametric bootstrapping was used to estimate bias-corrected CIs.

We did two prespecified subgroup analyses: belonging to Scheduled Caste, Scheduled Tribe, or other; and above and below the median of wealth measured as a score of 17 household assets. Evidence for any differential effects of the intervention on the primary and secondary outcomes by these subgroups was assessed by a treatment by subgroup interaction term. Where there was evidence of an interaction, the effects in the different subgroups were estimated directly from the regression model with the interaction term included. We explored the impact of direct exposure to the intervention through a per-protocol analysis comparing effects among women who reported participating in the intervention and those who did not. Statistical analyses were done using Stata (version 16).

We used tests before and after training to assess intervention workers' knowledge of nutrition and NSA, observation checklists to assess the quality of facilitation, and prespecified indicators to quantify intervention coverage by group as indicators of fidelity and quality of implementation. An in-depth process evaluation describing how contextual factors and intervention mechanisms could have influenced effects will be published separately.

We also did an economic evaluation. Total and average annual cost of the interventions was estimated from a programme provider perspective, including cost of setting up and implementing the interventions by the implementing partners. Costs were adjusted for inflation, discounted at 3% per year and converted to 2019 international dollar (INT$), using the purchasing power parity conversion factor for India (21·253)^29^ and UK (0·689).^29^ We employed cost-consequence analysis, the methodology for which is presented in detail elsewhere.^30^ A detailed analysis will be published separately.

### Role of the funding source

The study funder had no role in study design, data collection, data analysis, data interpretation, or writing of the report.

## Results

148 of 162 clusters assessed for eligibility were enrolled and randomly allocated to trial groups, giving 37 clusters per group. Of the 5427 households assessed for eligibility at baseline, 4480 mothers and 4473 spouses provided data. At endline, of 4792 households assessed for eligibility, 4291 mothers and 4287 spouses provided data (figure). The baseline survey was done between Nov 24, 2016, and Jan 24, 2017; clusters were randomised in two batches on December, 2016, and January, 2017; interventions were implemented from March 20, 2017, to Oct 31, 2019; and the endline survey was done between Nov 19, 2019, and Jan 12, 2020. The median households per village at baseline were 37 (IQR 33–39) for control, 35 (28–51) for AGRI, 34 (29–49) for AGRI-NUT, and 39 (IQR 31–52) for AGRI-NUT+PLA; and at 36 months were 28 (24–40) for control, 34 (27–48) for AGRI, 30 (26–45) for AGRI-NUT, and 36 (25–53) for AGRI-NUT+PLA.

**Figure:**
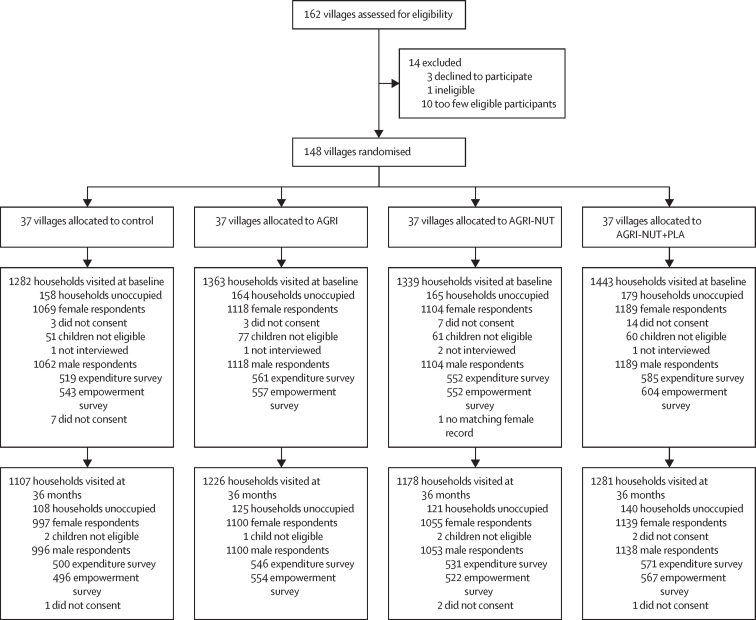
Trial profile NSA=nutrition-sensitive agriculture. AGRI=group assigned to NSA videos. AGRI-NUT=group assigned to NSA and nutrition-specific videos. AGRI-NUT+PLA=group assigned to NSA videos and a nutrition-specific participatory learning and action cycle meetings and videos.

Household characteristics at baseline were well balanced across groups (table 1). Households were predominantly of Scheduled Tribe (58%), with low levels of education (mothers' average: 6 years), and most (99%) owned some land, but only small parcels (81% of landowners owned <2·5 acres). Baseline measures of the primary, secondary, and other outcomes of interest are shown in table 2. Household characteristics at endline are in the appendix (p 17–18), and were similar at baseline and endline.

**Table 1:** Demographic characteristics of households at baseline

	**All groups (n=4480)**	**Control (n=1069)**	**AGRI (n=1118)**	**AGRI-NUT (n=1104)**	**AGRI-NUT+PLA (n=1189)**
**Proportion of Scheduled Caste or Scheduled Tribe households in the village**					
Low (<30%)	551 (12%)	143 (13%)	91 (8%)	182 (17%)	135 (11%)
Medium (30–70%)	2641 (59%)	665 (62%)	640 (57%)	620 (56%)	716 (60%)
High (>70%)	1288 (29%)	261 (25%)	387 (35%)	302 (27%)	338 (29%)
**Distance of village to nearest town**					
<10 km	824 (18%)	177 (17%)	218 (20%)	177 (16%)	252 (21%)
≥10 km	3656 (82%)	892 (83%)	900 (80%)	927 (84%)	937 (79%)
**District subdivision**					
Ghatagaon	1620 (36%)	298 (28%)	389 (35%)	454 (41%)	479 (40%)
Harichandanpur	1709 (38%)	449 (42%)	392 (35%)	413 (37%)	455 (38%)
Patna	672 (15%)	213 (20%)	170 (15%)	88 (8%)	201 (17%)
Keonjhar (Sadar)	479 (11%)	109 (10%)	167 (15%)	149 (14%)	54 (5%)
**Child's sex**					
Male	2264 (51%)	523 (49%)	561 (50%)	570 (52%)	610 (51%)
Female	2215 (49%)	546 (51%)	556 (50%)	534 (48%)	579 (49%)
Data missing	1 (0%)	0 (0%)	1 (0%)	0 (0%)	0 (0%)
**Child's age**					
Completed months	12·3 (6·5)	12·1 (6·5)	12·4 (6·4)	12·4 (6·6)	12·2 (6·4)
**Mother's age**					
Completed years	24·5 (4·0)	24·5 (4·1)	24·6 (4·0)	24·4 (3·8)	24·5 (4·2)
Data missing	13 (0%)	3 (0%)	1 (0%)	6 (1%)	3 (0%)
**Education of mother**					
Completed years	6·4 (4·5)	6·3 (4·5)	6·2 (4·6)	6·5 (4·4)	6·4 (4·5)
Data missing	3 (0%)	1 (0%)	1 (0%)	1 (0%)	0 (0%)
**Education of spouse**					
Completed years	7·2 (4·2)	7·4 (4·3)	7·1 (4·2)	7·2 (4·1)	7·3 (4·2)
Data missing	7 (0%)	2 (0%)	3 (0%)	1 (0%)	1 (0%)
**Household land ownership**					
Owns no land	38 (1%)	9 (1%)	11 (1%)	10 (1%)	8 (1%)
Owns land	4441 (99%)	1059 (99%)	1107 (99%)	1094 (99%)	1181 (99%)
Data missing	1 (0%)	1 (0%)	0 (0%)	0 (0%)	0 (0%)
Acres of land owned (if any)	1·10 (0·55–2·00)	1·03 (0·50–2·00)	1·04 (0·50–2·02)	1·20 (0·75–2·10)	1·10 (0·56–2·00)
>0 to <2·50 acres	3582 (81%)	876 (82%)	889 (80%)	859 (79%)	958 (81%)
2·50 to 5·00 acres	663 (15%)	133 (13%)	166 (15%)	189 (17%)	175 (15%)
>5·00 acres	184 (4%)	42 (4%)	52 (5%)	44 (4%)	46 (4%)
Acres of land owned unknown	12 (0%)	8 (1%)	0 (0%)	2 (0%)	2 (0%)
**Asset ownership**					
Sum of 17 assets	9·1 (2·6)	9·1 (2·6)	9·0 (2·6)	9·2 (2·6)	9·2 (2·6)
Data missing	10 (0%)	1 (0%)	3 (0%)	4 (0%)	2 (0%)
**Household size**					
Total number of household members	5·4 (2·1)	5·4 (2·7)	5·3 (1·8)	5·3 (1·7)	5·3 (1·8)
Data missing	3 (0%)	1 (0%)	1 (0%)	1 (0%)	0 (0%)
**Household composition**					
Contains male and female adults	4292 (96%)	1027 (96%)	1071 (96%)	1056 (96%)	1138 (96%)
Contains female only adults	178 (4%)	35 (3%)	47 (4%)	46 (4%)	50 (4%)
Data missing	10 (0%)	7 (1%)	0 (0%)	2 (0%)	1 1 (0%)

Data are n (%), mean (SD), or median (IQR). NSA=nutrition-sensitive agriculture. AGRI=group assigned to NSA videos. AGRI-NUT=group assigned to NSA and nutrition-specific videos. AGRI-NUT+PLA=group assigned to NSA videos and a nutrition-specific participatory learning and action cycle meetings and videos.

**Table 2:** Primary, secondary, and other outcome variables at baseline

	**Control**	**AGRI**	**AGRI-NUT**	**AGRI-NUT+PLA**
**Primary outcomes**				
Child minimum diet diversity* (ate ≥4 of 7 food groups in the last 24 h)	242/855 (28%)	259/925 (28%)	244/893 (27%)	286/982 (29%)
Maternal body-mass index, kg/m^2^†	19·2 (2·48), n=983	19·1 (2·57), n=1009	19·1 (2·45), n=998	19·2 (2·68), n=1116
**Secondary outcomes**				
Maternal minimum diet diversity (ate ≥5 of 10 food groups in the last 24 h)	243/1067 (23%)	203/1115 (18%)	247/1100 (22%)	263/1189 (22%)
Wasting (weight-for-height Z score SD <–2)	135/1049 (13%)	197/1113 (18%)	143/1095 (13%)	207/1173 (18%)
**Additional outcomes**				
Maternal low MUAC, <230 mm	482/1067 (45%)	506/1118 (45%)	524/1104 (47%)	560/1189 (47%)
Child low MUAC, <125 mm	65/856 (8%)	78/928 (8%)	72/897 (8%)	96/982 (10%)
Maternal haemoglobin (g/dL)‡	11·6 (1·18), n=1012	11·6 (1·11), n=1045	11·5 (1·15), n=1033	11·7 (1·21), n=1153
Child haemoglobin (g/dL)*	10·3 (1·22), n=850	10·2 (1·22), n=922	10·3 (1·21), n=891	10·4 (1·24), n=977
Child given minimum acceptable diet*	175/857 (20%)	181/928 (20%)	167/894 (19%)	186/982 (19%)
Women made ≥2 decisions in agriculture or health	923/1068 (86%)	979/1115 (88%)	944/1100 (86%)	1015/1189 (85%)
Women worked <10·5 h in the previous 24 h	371/1060 (35%)	457/1114 (41%)	436/1095 (40%)	526/1187 (44%)
Women achieving gender parity in agriculture§	207/500 (41%)	233/514 (45%)	230/518 (44%)	281/558 (50%)
Share of household expenditures spent on food¶	0·59 (0·18), n=519	0·61 (0·17), n=561	0·60 (0·18), n=552	0·59 (0·18), n=585
Per capita total daily household expenditure (INR)¶	17·6 (12·0 to 25·9), n=519	17·2 (11·6 to 24·0), n=561	17·8 (12·6 to 27·1), n=552	17·6 (12·3 to 26·9), n=585
Agricultural production diversity out of 10 food groups over 1 year	4·5 (1·57), n=1061	4·6 (1·52), n=1118	4·6 (1·63), n=1104	4·5 (1·56), n=1189
Total value of agricultural production over 1 year (INR)	4338 (2061 to 8195), n=1029	4492 (2344 to 8644), n=1079	4664 (2250 to 9930), n=1064	4328 (1981 to 8364), n=1162
Net value (total value minus input costs) of agriculture production over 1 year (INR)	1381 (−185 to 3920), n=1029	1655 (77 to 4715), n=1079	1500 (−301 to 5123), n=1064	1370 (−87 to 4056), n=1162

Data are n/N (%), mean (SD), or median (IQR). NSA=nutrition-sensitive agriculture. AGRI=group assigned to NSA videos. AGRI-NUT=group assigned to NSA and nutrition-specific videos. AGRI-NUT+PLA=group assigned to NSA videos and a nutrition-specific participatory learning and action cycle meetings and videos. MUAC=mid-upper arm circumference. INR=Indian Rupees.

* Includes children aged 6–23 months.

† Includes non-pregnant, non-postpartum women only.

‡ Includes non-pregnant women only.

§ Gender parity achieved when women have equal or higher empowerment scores than men; empowerment scores are calculated for men and women as weighted sums of five indicators: decision making, asset ownership, access to credit, group membership, and time use, measured in 50% of households; excludes female-only households.

¶ Valid measurements from expenditure questionnaire, administered on 50% of households.

Table 3 presents the adjusted intervention effects for the primary and secondary outcomes. The appendix (pp 5–6) shows results by group and stratification factors. We found higher proportions of children (aged 6–23 months) consuming at least four of seven food groups the previous day at endline in the AGRI-NUT (adjusted RR 1·19, 95% CI 1·03–1·37) and AGRI-NUT+PLA (1·27, 1·11–1·46) groups, each compared with the control group, and no effect in the AGRI group. The observed absolute difference between the proportion in the AGRI-NUT group and control of 6·4% was smaller than the effect size allowed for in the sample size calculations whereas the observed absolute difference between the AGRI-NUT+PLA and control was 10·1%. There was no difference in maternal BMI between any intervention groups and control at endline (adjusted mean differences AGRI −0·05, −0·34 to 0·24; AGRI-NUT 0·04, −0·26 to 0·33; AGRI-NUT+PLA −0·03, −0·30 to 0·23).

**Table 3:** Effect of interventions on the primary and secondary outcomes adjusted for baseline measures of the outcomes and stratification factors (caste and distance from nearest town)

	**Control**	**AGRI**	**AGRI-NUT**	**AGRI-NUT+PLA**	**AGRI *vs* control**		**AGRI-NUT *vs* control**		**AGRI-NUT+PLA *vs* control**	
					Adjusted relative risk (95% CI)	p value	Adjusted relative risk (95% CI)	p value	Adjusted relative risk (95% CI)	p value
**Primary outcomes**										
Child minimum diet diversity (ate ≥4 food groups)*	286/757 (38%)	325/822 (40%)	359/812 (44%)	413/863 (48%)	1·06 (0·91 to 1·23)	0·44	1·19 (1·03 to 1·37)	0·02	1·27 (1·11 to 1·46)	0·0006
Maternal body-mass index, kg/m^2^†	19·5 (2·81), n=923	19·3 (2·90), n=1014	19·4 (2·99), n=978	19·4 (2·92), n=1035	−0·05 (−0·34 to 0·24)	0·73	0·04 (−0·26 to 0·33)	0·80	−0·03 (−0·30 to 0·23)	0·81
**Secondary outcomes**										
Maternal minimum diet diversity (ate ≥5 food groups)	331/997 (33%)	396/1100 (36%)	402/1055 (38%)	479/1139 (42%)	1·21 (1·01 to 1·45)	0·043	1·16 (0·98 to 1·38)	0·077	1·30 (1·10 to 1·53)	0·0002
Wasting (weight-for-height Z score SD <–2)	133/986 (13%)	152/1096 (14%)	138/1052 (13%)	158/1133 (14%)	0·95 (0·73 to 1·24)	0·69	0·96 (0·72 to 1·29)	0·78	0·96 (0·73 to 1·26)	0·76

Data are mean (SD) or n/N (%) unless indicated otherwise. All denominators include children and mothers who participated in the endline survey and with valid measurements for each outcome. NSA=nutrition-sensitive agriculture. AGRI=group assigned to NSA videos. AGRI-NUT=group assigned to NSA and nutrition-specific videos. AGRI-NUT+PLA=group assigned to NSA videos and a nutrition-specific participatory learning and action cycle meetings and videos.

* Includes children 6–23 months only.

† Includes non-pregnant, non-postpartum women only.

At endline, higher proportions of mothers consumed at least five of ten food groups the previous day in the AGRI (adjusted RR 1·21, 95% CI 1·01–1·45) and AGRI-NUT+PLA (1·30, 1·10–1·53) groups, each compared with the control. There was borderline evidence of difference between the AGRI-NUT and control groups (adjusted RR 1·16, 0·98–1·38). Compared with control, the observed absolute differences in proportions at endline in AGRI (2·8%) and AGRI-NUT (4·9%) were smaller than the effect size hypothesised. The observed absolute difference in proportions between the AGRI-NUT+PLA and control (9·1%) was close to the effect size hypothesised in the sample size calculations for the primary outcome. There was no evidence of any difference in child wasting between interventions and control (adjusted RR AGRI 0·95, 0·73–1·24; AGRI-NUT 0·96, 0·72–1·29; AGRI-NUT+PLA 0·96, 0·73–1·26; table 3). The intracluster correlation coefficients were 0·03 (95% CI 0·01–0·05) for child minimum dietary diversity, 0·02 (0·01–0·04) for maternal BMI, 0·05 (0·03–0·09) for child wasting, and 0·05 (0·03–0·08) for maternal minimum dietary diversity. We found secular increases in child dietary diversity, maternal BMI, and maternal dietary diversity but not child wasting across all trial groups (appendix p 19).

Adjusted intervention effects for other outcomes are shown in table 4. At endline, child minimum acceptable diet was higher in AGRI-NUT (adjusted RR 1·19, 95% CI 1·02–1·41) and AGRI-NUT+PLA (1·30, 1·12–1·52) groups, each compared with control. There was no evidence of effect of the interventions on maternal or child MUAC or haemoglobin, women's time use, gender parity, household expenditures, or agricultural production diversity. In the AGRI group, compared with control, the proportion of women empowered in decision making was marginally higher (adjusted RR 1·05, 1·00–1·11). Both the total (INR7579, 95% CI 2211–16 298) and net annual (6825, 1781–15 412) values of agricultural production were higher. The appendix (pp 20–21) shows the effects of the interventions adjusted only for baseline measures of the outcomes.

**Table 4:** Effect of interventions on other outcomes adjusted for baseline measures of the outcomes and stratification factors (caste and distance from nearest town)

	**Control**	**AGRI**	**AGRI-NUT**	**AGRI-NUT+PLA**	**AGRI vs control**		**AGRI-NUT vs control**		**AGRI-NUT+PLA vs control**	
					Adjusted relative risk (95% CI)	p value	Adjusted relative risk (95% CI)	p value	Adjusted relative risk (95% CI)	p value
Maternal low MUAC <230 mm	445/989 (45%)	539/1096 (49%)	465/1051 (44%)	525/1134 (46%)	1·08 (0·96 to 1·23)	0·20	0·96 (0·85 to 1·08)	0·50	1·01 (0·90 to 1·13)	0·85
Child low MUAC <125 mm*	96/783 (12%)	128/861 (15%)	104/827 (13%)	110/893 (12%)	1·16 (0·85 to 1·57)	0·35	1·00 (0·76 to 1·31)	0·98	0·92 (0·66 to 1·28)	0·62
Maternal haemoglobin (g/dL)†	11·7 (1·30), n=974	11·6 (1·29), n=1077	11·6 (1·30), n=1033	11·6 (1·29), n=1111	0·02 (−0·15 to 0·19)	0·84	0·00 (−0·17 to 0·17)	0·98	−0·13 (−0·30 to 0·04)	0·15
Child haemoglobin (g/dL)*	10·1 (1·19), n=776	10·2 (1·29), n=857	10·1 (1·26), n=825	10·1 (1·25), n=883	0·11 (−0·07 to 0·28)	0·23	0·04 (−0·13 to 0·21)	0·68	−0·05 (−0·23 to 0·14)	0·62
Child minimum acceptable diet*	268/790 (34%)	290/862 (34%)	327/829 (39%)	384/895 (43%)	1·01 (0·86 to 1·19)	0·89	1·19 (1·02 to 1·41)	0·032	1·30 (1·12 to 1·52)	0·0008
Women made ≥2 decisions in agriculture or health	867/997 (87%)	1015/1100 (92%)	961/1055 (91%)	1002/1139 (88%)	1·05 (1·00 to 1·11)	0·038	1·05 (0·99 to 1·1)	0·12	1·02 (0·96 to 1·07)	0·55
Women worked <10·5 h in the previous 24 h	339/997 (34%)	339/1100 (31%)	332/1055 (31%)	357/1139 (31%)	0·79 (0·60 to 1·05)	0·11	0·84 (0·62 to 1·15)	0·28	0·76 (0·56 to 1·03)	0·082
Women achieving gender parity in agriculture‡	253/406 (62%)	283/439 (64%)	264/419 (63%)	294/465 (63%)	1·02 (0·91 to 1·13)	0·77	1·01 (0·90 to 1·13)	0·90	0·98 (0·87 to 1·10)	0·72
Share of household expenditures spent on food§	0·62 (0·17) n=500	0·63 (0·17) n=546	0·62 (0·17) n=531	0·63 (0·17) n=569	0·00 (−0·04 to 0·03)	0·80	−0·01 (−0·04 to 0·03)	0·69	0·01 (−0·02 to 0·04)	0·66
Per capita total daily household expenditure (INR)§¶	17·6 (12·9 to 25·7), n=500	18·2 (13·5 to 26·6), n=546	18·2 (13·4 to 26·7), n=531	18·2 (12·6 to 26·1), n=569	1·07 (−1·70 to 3·56)	0·43	0·83 (1·57 to 3·20)	0·50	2·39 (−0·35 to 5·40)	0·096
Agricultural production diversity out of 10 food groups over 1 year	4·1 (2·03), n=996	4·4 (2·03), n=1100	4·6 (1·95), n=1053	4·4 (2·02), n=1138	0·13 (−0·25 to 0·50)	0·51	0·37 (0·00 to 0·75)	0·052	0·31 (−0·07 to 0·69)	0·11
Total value of agricultural production over 1 year (INR)¶	16 696 (6312 to 34 400), n=996	19 465 (8475 to 37 648), n=1100	19 668 (9229 to 38 368), n=1053	17 378 (7395 to 34 683), n=1138	7579 (2211 to 16 298)	0·03	−323 (−9442 to 8295)	0·94	4922 (−163 to 13 407)	0·15
Net value (total value minus input costs) of agriculture production over 1 year (INR)¶	10 048 (2810 to 24 241), n=996	13 489 (4405 to 28 118), n=1100	13 581 (4792 to 27 869), n=1053	11 885 (3528 to 25 779), n=1138	6825 (1781 to 15 412)	0·045	−197 (−9218 to 7909)	0·96	5324 (857 to 13 817)	0·11

Data are mean (SD), n/N (%), or median (IQR), unless indicated otherwise. All denominators are valid responses from the endline survey. NSA=nutrition-sensitive agriculture. AGRI=group assigned to NSA videos. AGRI-NUT=group assigned to NSA and nutrition-specific videos. AGRI-NUT+PLA=group assigned to NSA videos and a nutrition-specific participatory learning and action cycle meetings and videos. MUAC=mid-upper arm circumference.

* Includes children aged 6–23 months only.

† Includes non-pregnant women only.

‡ Gender parity achieved when women have equal or higher empowerment scores than men; empowerment scores are calculated for men and women as weighted sums of five indicators: decision making, asset ownership, access to credit, group membership, and time use; measured in 50% of households, excludes female-only households.

§ Measured in 50% of households.

¶ Confidence intervals are bias corrected estimated using non-parametric bootstrapping.

There was no evidence of differential effects of the interventions on the prespecified subgroups: caste and household wealth (data not shown). Results from the per-protocol analysis (appendix p 22) support the effects observed in the intention-to-treat analysis and suggest that exposure to the interventions is important.

Our indicators of fidelity and exposure suggest high-quality implementation in all intervention groups. Training improved group facilitators' and video producers' knowledge before the start of interventions, with mean knowledge scores for 82 facilitators and video producers increasing from 14·4 to 22·6 out of 23 (SD 0·6) for NSA, and 12·7 to 18·9 out of 20 (SD 0·2) for maternal and child nutrition. Table 5 reports data on the quality of implementation and exposure to interventions. Over 97% of intervention events occurred as planned, with little difference between groups. Internal quality monitoring checks done in the 6 months before endline suggested all 148 observed video disseminations were high quality, as were four of seven observed PLA meetings (data not shown). At endline, in all intervention groups, half of all mothers said they were active self-help group members, and half or more reported exposure to a video or PLA meeting in the 6 months before the endline, with slightly higher proportions in the AGRI-NUT (58%) and AGRI-NUT+PLA (55%) groups than in the AGRI (50%) group.

**Table 5:** Process indicators on implementation fidelity and exposure to interventions

		**Control**	**AGRI**	**AGRI-NUT**	**AGRI-NUT+PLA**
**Quality of implementation: internal monitoring data**					
Number of events (video or PLA meetings) achieved in the 32-month implementation period		NA	16 551/16 996 (97%)	15 790/16 156 (98%)	13 986/14 162 (99%)
Structured observations of video dissemination quality in the last 6 months and quality score achieved*					
	Grade A	NA	51/51 (100%)	57/57 (100%)	40/40 (100%)
	Grade B	NA	0/51 (0%)	0/57 (0%)	0/40 (0%)
	Grade C	NA	0/51 (0%)	0/57 (0%)	0/40 (0%)
**Exposure to interventions: endline survey data**					
Active self-help group members		458/997 (46%)	530/1100 (48%)	537/1055 (51%)	546/1139 (48%)
Went to any video dissemination or PLA meeting in last 6 months		21/997 (2%)	548/1100 (50%)	607/1055 (58%)	632/1139 (55%)
Mean number of events (video or PLA meetings) attended in last 6 months; range 0–11		0·1 (0·8), n=997	3·3 (4·0), n=1100	4·4 (4·5), n=1055	3·6 (3·9), n=1139

Data are n/N (%) or mean (SD). NSA=nutrition-sensitive agriculture. AGRI=group assigned to NSA videos. AGRI-NUT=group assigned to NSA and nutrition-specific videos. AGRI-NUT+PLA=group assigned to NSA videos and nutrition-specific participatory learning and action (PLA) cycle meetings and videos.

* The assessor scored eight parameters relating to the facilitator's ability as 0 (poor), 1 (good), or 2 (very good), for grades C, B, and A, respectively. Parameters were handling and setting up equipment; preparing the meeting venue; introducing the video topic; pausing the video during screening to check understanding and answer questions; encouraging the adoption of practices shown in the video; summarising the video; answering questions requiring subject knowledge; and filling out a form about the dissemination. Video disseminations scoring a total of 12 points or more achieved grade A (high quality), 6–11 points achieved grade B, and 0–7 points achieved grade C.

Total costs of the intervention groups were INT$639 541 for AGRI, 789 243 for AGRI-NUT, and 986 865 for AGRI-NUT+PLA. Average annual costs were 187 182 for AGRI, 230 998 for AGRI-NUT, and 288 838 for AGRI-NUT+PLA. Costs per pregnant woman or mother of children (aged <2 years) covered were 146 for AGRI, 182 for AGRI-NUT, and 199 for AGRI-NUT+PLA. Costs per person covered (all ages) were 16 for AGRI, 20 for AGRI-NUT, and 21 for AGRI-NUT+PLA. Staff costs, on average, accounted for 66% of the total costs in each intervention group, followed by other recurrent costs (26%). Costs for the activities conducted in the start-up period (9 months) accounted for 21% of total costs. The appendix (p 23) provides breakdown interventions' costs by component and unit costs of delivery for each intervention.

## Discussion

To our knowledge, our study is the first RCT to evaluate the effects of NSA interventions on maternal and child nutrition in India. The trial tested the impact of three NSA interventions using participatory videos and women's group meetings, each compared with the control, on maternal and children's dietary diversity, maternal BMI, and child wasting. We found that interventions improved children's and mothers' dietary diversity and children's minimum acceptable diet. None of the interventions affected maternal BMI or child wasting.

We found positive effects of AGRI-NUT and AGRI-NUT+PLA on child minimum dietary diversity (AGRI-NUT and AGRI-NUT+PLA groups: 19% and 27% relative increase in the chance of children meeting minimum dietary diversity than control, respectively), in addition to secular relative improvement of 33% from baseline to endline. NSA trials from Ghana, Malawi, and Nepal have also shown positive, significant impacts on child minimum dietary diversity.^31^, ^32^, ^33^, ^34^ However, others in Ethiopia,^35^ Zambia,^36^ Nepal,^37^ and Burkina Faso^28^ showed no impact.

In our trial, women had a relative increase of 21% (AGRI), 16% (AGRI-NUT), and 30% (AGRI-NUT+PLA) in the chance of meeting minimum dietary diversity than control, albeit borderline statistical significance in AGRI-NUT. Only two other NSA trials have measured effects on maternal dietary diversity (from Burkina Faso and Zambia) and neither had a significant impact.^38^, ^39^

These results show that making agriculture interventions nutrition-sensitive can improve diets. Although our study was not designed to detect differences between interventions, the largest observed effect sizes for dietary diversity were found in the AGRI-NUT+PLA group versus control. This result suggests that enhancing participatory components using a PLA cycle could accelerate NSA intervention improvements in diet quality. The participatory nature of interventions might have created an enabling environment for women to adopt new dietary practices through peer support, building women's confidence, problem solving, and collective action.

Similar to our study, trials from Burkina Faso,^38^ Nepal,^33^ and Cambodia^40^ found no effects of NSA interventions on mean maternal BMI, although small reductions in the prevalence of maternal underweight were found in Burkina Faso^38^ and Nepal.^33^ Improvements in women's dietary diversity, an indicator of micronutrient adequacy,^23^ could be insufficient to change maternal anthropometry; additional increases in energy intake, reduction in energy expenditure, or both might be required. Furthermore, the secular increase in BMI by 0·25 kg/m^2^ from baseline to endline might have attenuated our ability to detect a difference of 0·3 kg/m^2^ as hypothesised for this trial. Secular state-wide improvements in maternal BMI might be explained by expansion and strengthening of health services through National Health Mission, and the Mamata Scheme providing conditional cash transfers^41^ to pregnant women. Coverage of take-home rations in Odisha increased for pregnant women (44·6% to 60·6%) and breastfeeding women (39·8% to 76·5%) from 2006 to 2016, during which the proportion of underweight women also declined by 15 percentage points.^3^, ^42^

Our study, like all six other NSA trials that measured it, found no impact on child wasting.^32^, ^33^, ^35^, ^36^, ^40^, ^43^ Much of the nutrition-specific evidence on child wasting interventions focuses on treatment; effective prevention strategies are needed.^44^ A recent study^45^ found that wasting incidence peaks from birth to 3 months, highlighting the importance of preventive interventions starting in the preconception period, pregnancy, and early infancy.

We found some improvements in agricultural production and women's decision making, but effects were not consistent across intervention groups, and we found no effects on food expenditures or women's time use. Although these outcomes are heterogeneously measured, other NSA interventions have also empowered women^36^, ^38^ and improved production diversity.^32^, ^39^ More disaggregated investigation into production of key crops, food purchasing, and household allocative behaviour might be required to explain the dietary improvements. Furthermore, we saw large secular improvements in gender parity and value of agriculture, perhaps reflecting Odisha's progress on many fronts,^46^, ^47^ including economic and agricultural growth, investment in self-help groups, and agricultural extension through Odisha's Livelihood Mission. Lack of differential effects by subgroups suggests our interventions had an equitable impact.

UPAVAN interventions' costs ranged from INT$146 to 199 per pregnant woman or mother of a child aged under 2 years. These are low compared with other nutrition or health interventions with an agriculture component, such as community or homestead food production^48^ and biofortification.^49^ Cost per beneficiary in these studies range from 120 to 3000 in 2019.

UPAVAN was an observer-blinded cluster RCT of complex interventions done with equitable partnerships. We included three key features highlighted in a recent systematic review of women's groups on maternal and child health:^13^ high implementation fidelity; a focus on problem solving and capacity building; and intergenerational participation, by including grandparents and adolescents. Strong feedback loops ensured the interventions remained demand-driven and relevant.^20^ Our intervention packages were derived from prevailing policy interventions and are amenable to scale up.

Our trial has limitations. Self-reported outcomes can be biased, although we used age-appropriate, validated, globally recommended instruments. Dietary scores do not capture diversity within food groups but do indicate micronutrient adequacy at the population level.^23^, ^24^ Empowerment, expenditures, and agricultural yields are complex constructs and could contain measurement error. Our control and intervention groups were geographically close, although we did not find control group contamination in terms of participation. Finally, generalising results from RCTs requires additional information about context, mechanisms, outcomes, and interactions between these, which our process evaluation will explore.^50^

UPAVAN interventions contain approaches that already exist at scale in several Indian states, albeit with little integration of nutrition objectives or convergence at a community level.^46^ These include participatory agriculture videos that reach over 1·5 million Indian farmers through women's self-help groups supported by the National and State Livelihood Missions, nutrition-specific videos delivered through women's groups to over 1 million women by State Departments of Health and Family Welfare or Department of Women and Child Development,^51^ and PLA meetings to improve maternal and child health and nutrition by the National Health Mission, as implemented at scale with incentivised community health workers in Jharkhand and Madhya Pradesh.^52^ These initiatives could be optimised to accelerate improvements in maternal and child diet quality by incorporating nutrition objectives in agricultural videos, coupling this with nutrition-specific behaviour change interventions such as the videos and PLA meetings, and ensuring convergence of programmes from relevant sectors to tackle the key determinants of nutrition, as recommended in the Convergent Nutrition Action Plans under the National Nutrition Mission, Prime Minister's Overarching Scheme for Holistic Nutrition Abhiyaan.^53^

Participatory interventions of women's groups using combinations of NSA videos, nutrition-specific videos, and a nutrition-specific PLA cycle can improve maternal and child diet quality in rural settings in India. These approaches have been implemented separately in different contexts and could be scaled up together to optimise effects on diets. Scaling up such approaches is, however, unlikely to be sufficient to improve child wasting. Prevention of child wasting is likely to require long-term investments in equitable, intergenerational, and convergent nutrition-sensitive multisectoral approaches to ensure macronutrient and micronutrient dietary adequacy, infection prevention, and access to child care and health care.

## Data sharing

All the individual participant data collected during the trial will be available immediately after publication with no end date after de-identification. Data will be available from the LSHTM Data Compass, an open-assess institutional research data repository.

## Declaration of interests

We declare no competing interests.
