# Supplementary material for: Effect of nutrition-sensitive agriculture interventions with participatory videos and women's group meetings on maternal and child nutritional outcomes in rural Odisha, India (UPAVAN trial): a four-arm, observer-blind, cluster-randomised controlled trial
Source: Lancet Planet Health. 2021 Mar 31;5(5):e263–76. doi: 10.1016/S2542-5196(21)00001-2 (PMC8099729; doi:10.1016/S2542-5196(21)00001-2)
Supplement: Supplementary appendix [file mmc1.pdf]

THE LANCET
Planetary Health

Supplementary appendix

This appendix formed part of the original submission and has been peer reviewed. We post it as supplied by the authors.

Supplement to: Kadiyala S, Harris-Fry H, Pradhan R, et al. Effect of nutrition-sensitive agriculture interventions with participatory videos and women's group meetings on maternal and child nutritional outcomes in rural Odisha, India (UPAVAN trial): a four-arm, observer-blind, cluster-randomised controlled trial. *Lancet Planet Health* 2021; published online March 31. http://dx.doi.org/10.1016/S2542-5196(21)00001-2.

Contents

Supplementary Figure 1 - Map of study site 1

Supplementary Figure 2 - UPAVAN Intervention arms 2

Supplementary Figure 3 - UPAVAN's Theory of Change 3

Supplementary Figure 4 - Illustration of how the four phases of the Participatory Learning and Action cycle feed into the participatory video approach in groups in AGRI-NUT+PLA 4

Supplementary Figure 5 - Forest plot of primary and secondary outcomes by the stratification criteria 5

Supplementary Table S1 - Search topics and terms used in the literature search of nutrition-sensitive agriculture randomised controlled trials, published from January 2017 – April 2020, adapted from Ruel, *et al.* 2018[1] 7

Supplementary Table S2 - Inclusion and exclusion criteria used for review of nutrition-sensitive agricultural randomised controlled trials, adapted from Ruel, et al. 2018[1] 8

Supplementary Table S3 - Summary of findings from nutrition-sensitive agriculture randomised controlled trials reviewed from January 2017-April 2020, update to Ruel, et al. 2018[1] 9

Supplementary Table S4 - Trial Indicators 15

Supplementary Table S5 - Demographic characteristics of households at endline 17

Supplementary Table S6 - Changes between baseline and endline in the primary, secondary, and other outcomes across all trial arms adjusted for the stratification factors (caste and distance from nearest town), after accounting for any differences attributable to the interventions 19

Supplementary Table S7 - Effect of interventions only adjusted for baseline measures of the outcome 20

Supplementary Table S8 - Per protocol analysis adjusted for baseline measures of the outcomes and stratification factors (caste and distance from nearest town) 22

Supplementary Table S9a - Components of total costs by intervention arm 23

Supplementary Table S9b - Cost description and unit costs of the UPAVAN interventions (2019 INT$) 23

Supplementary Figure 1 - Map of study site

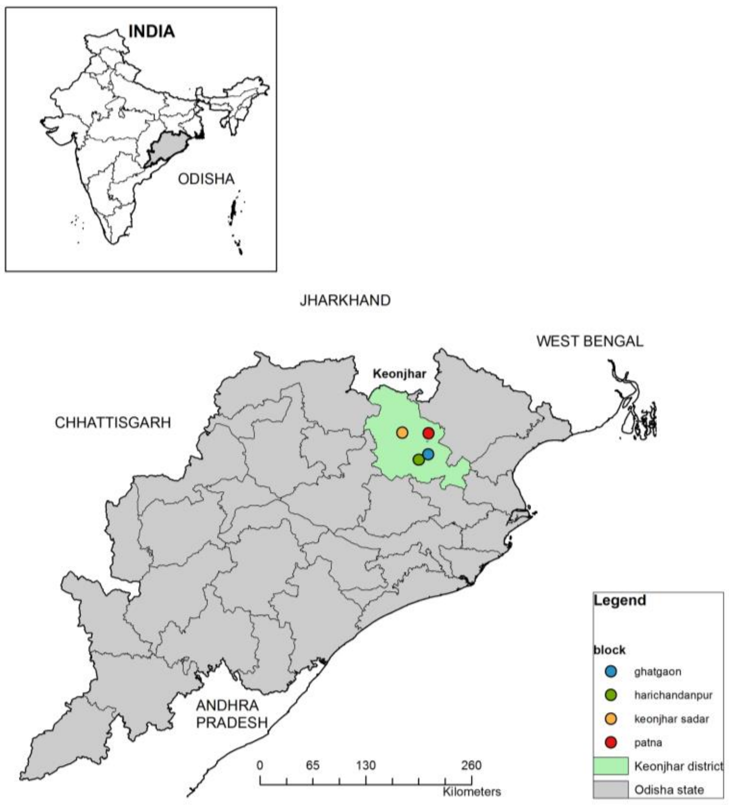

Source: Reproduced with permission from Kadiyala S, Prost A, Harris-Fry H, *et al.* Upscaling Participatory Action and Videos for Agriculture and Nutrition (UPAVAN) trial comparing three variants of a nutrition-sensitive agricultural extension intervention to improve maternal and child nutritional outcomes in rural Odisha, India: study protocol for a cluster randomised controlled trial. *Trials* 2018; **19**: 176.

Supplementary Figure 2 - UPAVAN Intervention arms

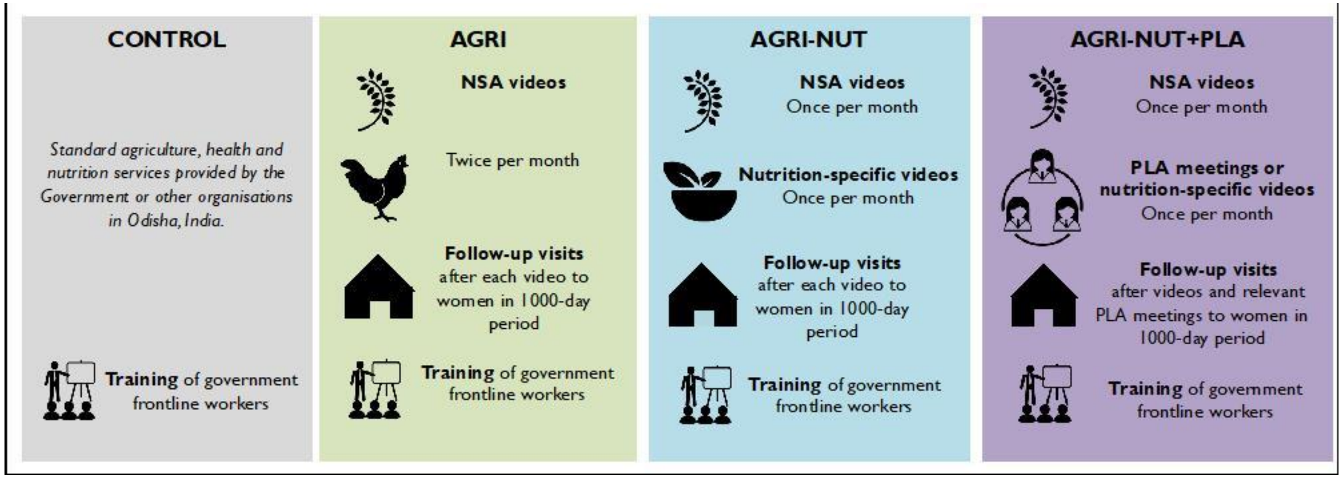

Source: Reproduced with permission from Kadiyala S, Prost A, Harris-Fry H, *et al.* Upscaling Participatory Action and Videos for Agriculture and Nutrition (UPAVAN) trial comparing three variants of a nutrition-sensitive agricultural extension intervention to improve maternal and child nutritional outcomes in rural Odisha, India: study protocol for a cluster randomised controlled trial. *Trials* 2018; **19**: 176.

Supplementary Figure 3 - UPAVAN's Theory of Change

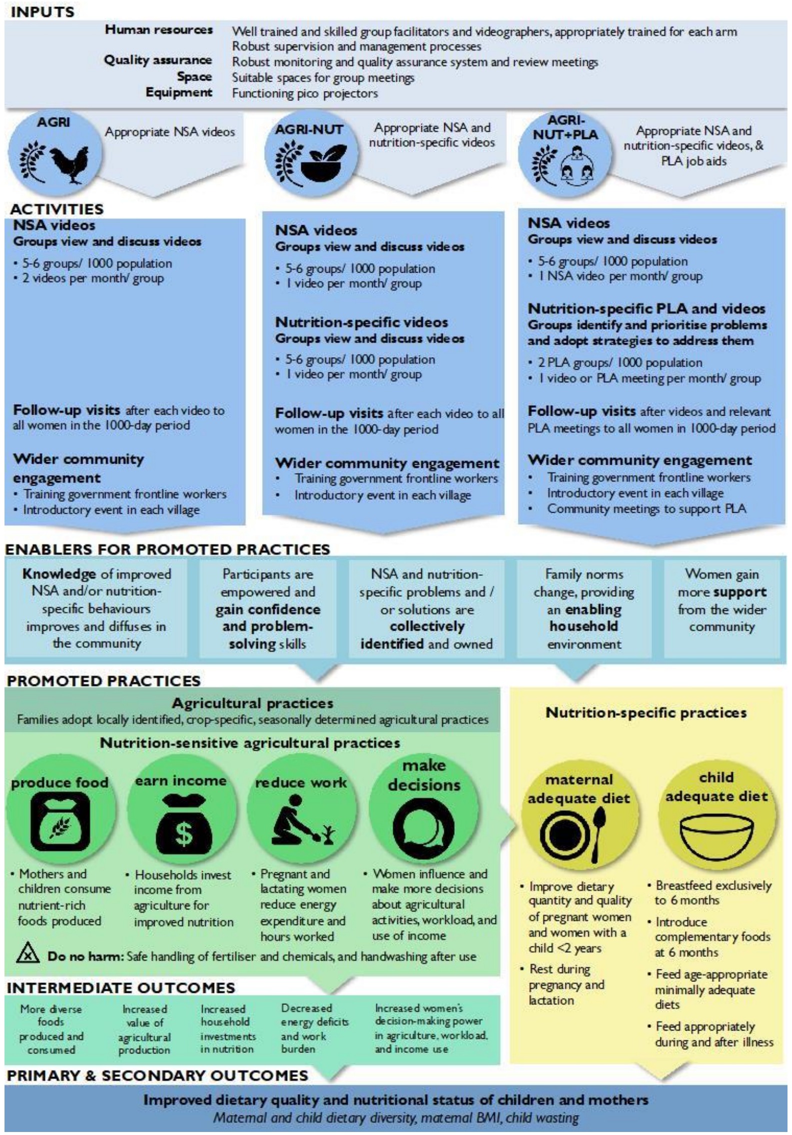

Source: Reproduced with permission from Kadiyala S, Prost A, Harris-Fry H, *et al.* Upscaling Participatory Action and Videos for Agriculture and Nutrition (UPAVAN) trial comparing three variants of a nutrition-sensitive agricultural extension intervention to improve maternal and child nutritional outcomes in rural Odisha, India: study protocol for a cluster randomised controlled trial. *Trials* 2018; **19**: 176.

Supplementary Figure 4 - Illustration of how the four phases of the Participatory Learning and Action cycle feed into the participatory video approach in groups in AGRI-NUT+PLA

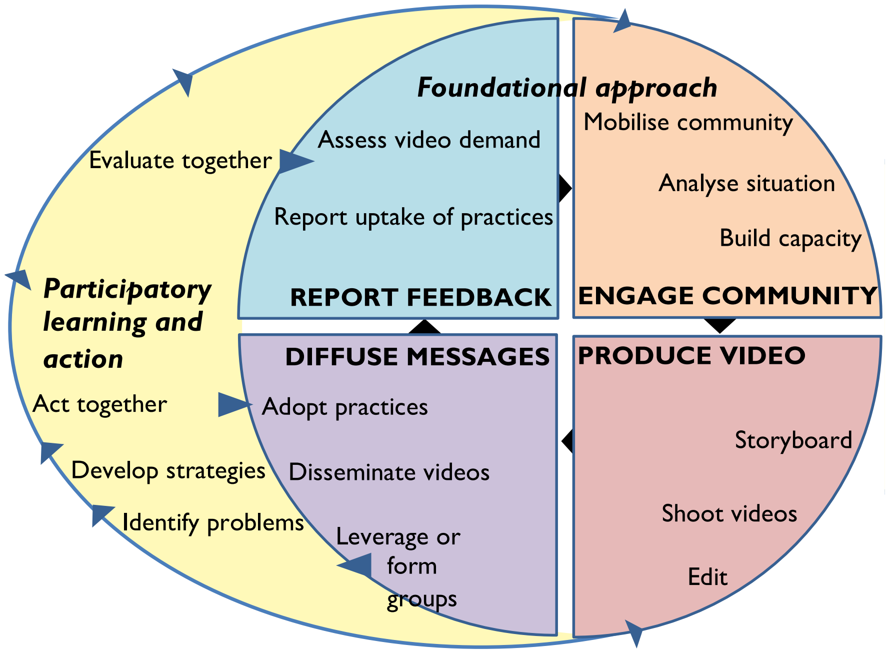

Source: Reproduced with permission from Kadiyala S, Prost A, Harris-Fry H, *et al.* Upscaling Participatory Action and Videos for Agriculture and Nutrition (UPAVAN) trial comparing three variants of a nutrition-sensitive agricultural extension intervention to improve maternal and child nutritional outcomes in rural Odisha, India: study protocol for a cluster randomised controlled trial. *Trials* 2018; **19**: 176.

Supplementary Figure 5 - Forest plot of primary and secondary outcomes by the stratification criteria

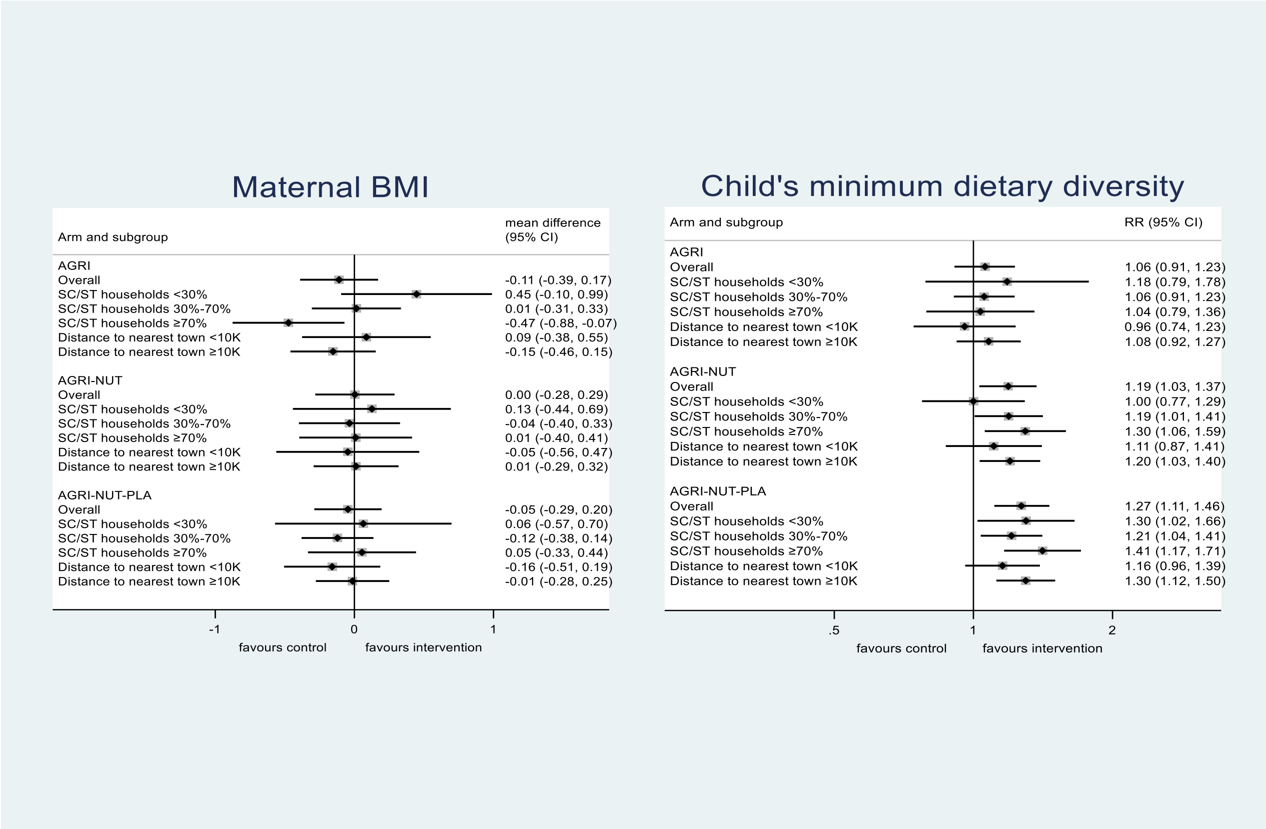

Abbreviations: BMI=body mass index. RR=relative risk. SC=scheduled caste. ST=scheduled tribe.

Supplementary Figure 5 - Forest plot of primary and secondary outcomes by the stratification criteria

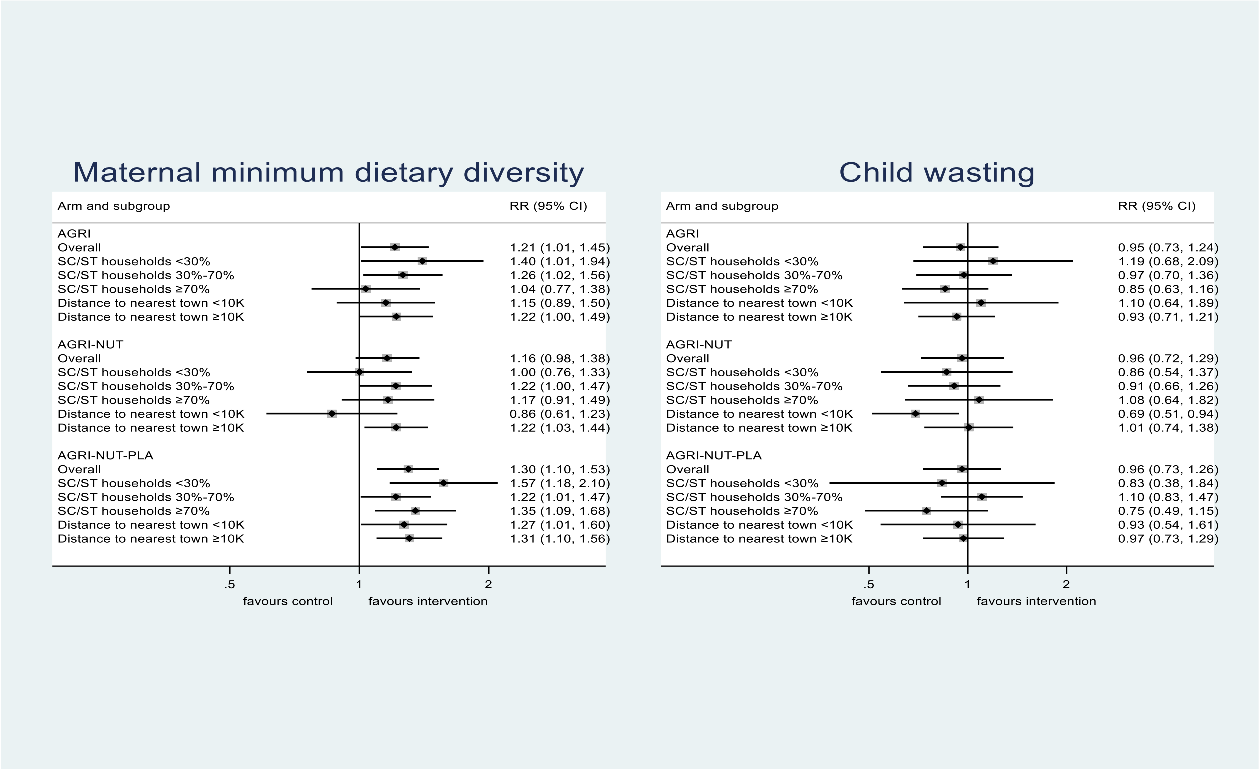

Abbreviations: RR=relative risk. SC=scheduled caste. ST=scheduled tribe.

Supplementary Table S1 - Search topics and terms used in the literature search of nutrition-sensitive agriculture randomised controlled trials, published from January 2017 – April 2020, adapted from Ruel, *et al.* 2018[1]

Topic	Search terms
**1. Nutrition**	"nutrition* outcome", "nutrition* status" "diet* diversity" "diet* diversification" micronutrient* anthropom*
**2. Biofortification**	biofortif* OR bio-fortif* OR "harvestplus" OR "harvest plus"
**3. Homestead production**	"homestead production" "homestead food production" "home garden" "homestead garden" "home gardening"
**4. Livestock and dairy**	"livestock* programs" "livestock* production" "livestock* ownership" "dairy* production""dairy* program"
**5. Agriculture Extension**	"agricultur* extension"
**6. Irrigation**	Irrigation AND impact
**7. Aquaculture**	Aquaculture OR fisheries or fishpond
**8. Value chains**	("value chain" OR value-chain) AND (nutrition OR diet)
**9. Nutrition-sensitive agriculture**	("nutrition-sensitive" OR "nutrition sensitive") AND agriculture
**10. RCT**	RCT OR "randomised controlled trial" OR "randomised control trial"
**Search string = 1 AND (2 OR 3 OR 4 OR 5 OR 6 OR 7 OR 8 OR 9) AND 10**	

[1]Ruel MT, Quisumbing AR, Balagamwala M. Nutrition-sensitive agriculture: What have we learned so far? *Glob Food Sec* 2018; **17**: 128–53.

Supplementary Table S2 - Inclusion and exclusion criteria used for review of nutrition-sensitive agricultural randomised controlled trials, adapted from Ruel, et al. 2018[1]

Criterion	Include	Exclude
**Publication type**	Peer reviewed articles, published working papers and abstracts, and online reports	Unpublished abstracts, reports, and briefs
**Publication years**	January 2017-April 2020	Earlier than January 2017
**Study type**	Randomised controlled trials	Non-randomised controlled trials
**Agriculture**	Homestead production/ home gardening<br>Irrigation<br>Value chains<br>Agriculture extension	Food systems<br>Food safety<br>Biofortification
**Nutrition**	Anthropometry (weight-for-height z-score, height-for-age z-score, weight-for-age z-score, stunting, wasting, underweight, mid-upper arm circumference, weight, height, birth weight<br>Anaemia/ haemoglobin<br>Diet/ diet diversity<br>Macronutrient intake (proteins, fats, carbohydrates)<br>Micronutrient intake (Vitamin A, iodine, zinc, folic acid)<br>Micronutrient status	Health outcomes not directly related to nutrition (such as delivery complications)
**Location**	Low- and middle-income countries	High-income countries
**Other**		Animal/ plant outcomes

[1]Ruel MT, Quisumbing AR, Balagamwala M. Nutrition-sensitive agriculture: What have we learned so far? *Glob Food Sec* 2018; **17**: 128–53.

Supplementary Table S3 - Summary of findings from nutrition-sensitive agriculture randomised controlled trials reviewed from January 2017-April 2020, update to Ruel, et al. 2018[1]

Author(s), year, study location	Evaluation design	Intervention	Outcomes measured	Findings	Authors' own conclusions
Gelli, et al. 2020, Malawi[2]	• Longitudinal cluster RCT<br>• 60 community-based childcare centres selected at random from a pool of 235 in 47 primary school clusters.<br>• Childcare centres randomly assigned to treatment or control. Both groups supported by an early childhood development program; treatment group had an additional agriculture and nutrition intervention.<br>• Two cross-sectional surveys, one year apart (Dec 2015 and 2016)<br>• Children 3-6 y at baseline (control: n=662, intervention: n=654)	*Nutrition:* Behaviour change communication activities and education on infant and young child feeding, food selection, preparation, and hygiene<br><br>*Agriculture:*<br>Activities included input provision (i.e., 10 chicks per household and seeds) and training on production of nutritious food (animal-source foods, vitamin A-rich staples)	Children's probability of adequacy of intakes of multiple micronutrients, measured using 24-h dietary recalls.	Positive impact on the probability of adequacy of intake of:<br>• Vitamin A (DID 9 pp, SE 3 pp)<br>• Vitamin C (DID 14 pp, SE 3 pp),<br>• Riboflavin (DID 11 pp, SE 3 pp),<br>• Zinc (DID 8 pp, SE 3 pp), Mean probability of adequacy of 11 micronutrients (DID 5 pp, SE 1 pp)<br><br>No impact on the probability of adequacy of intake of: thiamin, niacin, vitamin B-6, folate, vitamin B-12, calcium, or iron.	Using a preschool platform to implement an NSA behaviour change communication intervention is an effective strategy to improve the adequacy of micronutrient intake of preschool children in food-insecure settings. Community contributions may provide a sustainable option for governments in low- and middle-income countries.
Gelli, et al. 2018. Malawi[3]	• As Gelli, et al. [2]<br>• A secondary reference group included children aged 6–24 mo at baseline (younger sibling group) living in households with ≥1 other child in the pre-schooler group (3-6 y)	As Gelli, et al. [2]	Children's:<br>• Mean food groups consumed of 12 food groups and nutrient intake (3-6 y) and minimum dietary diversity (≥4 of 7 food groups) (6-23 months) assessed by multi-pass 24-h recall method.<br>• Anthropometry (height-for-age, weight-for-age, and weight-for-height Z scores)<br><br>Caregiver infant and young child feeding knowledge and practices<br><br>Crop production diversity (count of number of foods groups out	Children 3-6 y:<br>• Greater increases in mean food groups consumed (intervention vs control DID 0.36; $p<0.0001$) and nutrient intake.<br>• No impacts on anthropometric measures<br><br>Children 6-23 months (secondary reference group):<br>• Intervention had significant effects on height-for-age z-scores (DID 0.44; $p<0.05$), stunting prevalence (-17pp; $p<0.05$). No impact on weight-for-age and weight-for-height z-scores	Implementing an integrated agriculture and nutrition intervention through early childhood development platforms increase child dietary diversity and nutrient intake and reduce stunting among younger siblings of targeted pre-schoolers.<br><br>The plausibility of the impact on growth in younger siblings is supported by improvements along program impact pathways, including production of nutritious food and minimum dietary diversity.

Author(s), year, study location	Evaluation design	Intervention	Outcomes measured	Findings	Authors' own conclusions
			of 12 food groups) and crop production variety (total count of number of individual crops) cultivated in the previous 12 months.	• Intervention increased the proportion achieving minimum dietary diversity (11pp difference; p<0.05)<br><br>• Significant increase in crop production diversity (intervention vs control DID impact 0.71, p<0.001) and crop production variety (intervention vs control DID impact 2.14, p<0.001)	
Kim, et al. 2018. Ethiopia[4]	• Longitudinal cluster RCT<br><br>• 20 rural woredas were randomly assigned to the intensive programme (interpersonal communication, NSA activities, community mobilisation, and mass media) or non-intensive programme (standard agriculture extension services and less intensive community mobilisation and mass media)<br><br>• Repeat cross-sectional household survey at baseline (2015) and endline (2017)<br><br>Children aged 6-23 months at baseline (intensive: n=1328, non-intensive: n=1318)	*Nutrition:* Behaviour change communication on infant and young child feeding-focused counselling, standard nutrition counselling, and food demonstrations, and mass media conveying infant and young child feeding messages<br><br>*Agriculture:* Promotion of NSA (i.e., prioritising vegetables from home garden to children <2 years)	Children's:<br><br>• Minimum dietary diversity (≥4 of 7 food groups), minimum meal frequency, minimum acceptable diet<br><br>• Anthropometry (height-for-age, weight-for-age, and weight-for-height)<br><br>Maternal complementary feeding knowledge	• Statistically marginal improvements for minimal dietary diversity and minimal acceptable diet in intensive vs non-intensive arms (6.6%; p=0.08 and 5.5%; p=0.07, respectively. No impact on minimum meal frequency.<br><br>• Positive impact on maternal complementary feeding knowledge (intensive vs non-intensive DID impact 0.7; p<0.001)<br><br>• Positive impact on stunting prevalence (intensive vs non-intensive DID impact -5.6 pp; p<0.05)<br><br>• No impact on the prevalence of wasting and underweight, height-for-age, weight-for-age, and weight-for-height z-scores)	Delivery of social behaviour change communication interventions using multiple platforms is an effective and feasible way to reduce stunting and improve complementary feeding practices within a two-year period.<br><br>Continued efforts are required to expand intervention coverage and improve complementary feeding practices across Ethiopia.
Marquis, et al. 2018, Ghana[5]	• Longitudinal cluster RCT<br><br>• 16 clusters randomly assigned to intervention (nutrition behaviour change communication and	*Nutrition:* Group behaviour change communication on nutrition focusing on dietary diversity. Community nutrition education (food demonstrations and infant	Children's:<br><br>• Egg consumption and minimum dietary diversity (≥4 of 7 food groups) (6-	• Positive impact on child minimum dietary diversity (adjusted OR treatment compared to control 1.65, p<0.05). No impact on egg consumption.	The one-year intervention had positive impacts on child minimum dietary diversity and mean height-for-age and weight-for-age. The intervention mitigated the

Author(s), year, study location	Evaluation design	Intervention	Outcomes measured	Findings	Authors' own conclusions
	agricultural inputs and training) or control.<br>• Cross-sectional surveys at baseline and endline (12-months later)<br>• Sample of 500 mother-infant (0-18 mo) dyads (intervention n=278, control n=213)	and young child feeding practices)<br>*Agriculture:* Livestock and home garden training including coop construction using local materials and caring for poultry. Distribution of inputs (e.g. seeds and vines and facilitated egg sales)	23 months) using 24-h recall<br>• Anthropometry (height-for-age, weight-for-age, and weight-for-height)	• Positive impact on mean height-for-age and weight-for-age (intervention vs. control β=0.22, p<0.01); β=0.15, p<0.05, respectively). No impact on weight-for-height.	decline in linear growth that occurs in late infancy in Ghanaian communities. The height-for-age decline in the intervention groups was <50% of the national cross-sectional difference.
Miller, et al. 2020, Nepal[6]	• Longitudinal cluster RCT<br>• Three groups of villages randomly assigned to; (i) full package (community development activities via women's groups); (ii) partial package (livestock training and nutrition education; or control (no intervention).<br>• Five repeat cross-sectional surveys over 36 months<br>• Households with children aged 1-60 mo at baseline (control: n=416; full package: n=370; partial package: n=366)	*Nutrition*: Behaviour change communication on infant and young child feeding, nutrition for expecting mothers, and hygiene<br>*Agriculture:* Livestock management training<br>*Social capital development*: poverty alleviation, citizen empowerment, and community development	Children's:<br>• Minimum dietary diversity (≥4 of 7 food groups)<br>• Anthropometry (height-for-age, weight-for-age, and weight-for-height, and mid-upper-arm circumference)	• No impact on child minimum dietary diversity comparing either interventions to the control.<br>• Positive impact on height-for-age z-scores in full package and partial package vs control (β=0.29, p<0.01; β=0.13, p<0.05, respectively).<br>• Positive impact on mid-upper arm circumference z-scores in partial package vs control (β=0.31, p<0.01). No impact comparing full package to control.<br>• Positive impact on weight-for-age z-scores in full package vs control (β=0.11, p<0.01). No impact comparing partial package to control.<br>• No impact on weight-for-height z-scores for either interventions compared to control.	Comprehensive multisectoral intervention was more successful at improving key growth indicators in children. Provision of nutrition education and training in livestock management alone had a limited effect on these outcomes.
Michaux, et al. 2019, Cambodia[7]	• Longitudinal cluster RCT<br>• 10 households in 90 clusters were randomly assigned to (1) enhanced	*Nutrition:* Behaviour change communication on nutrition and hygiene focusing on optimal nutrition for women	Measured in women and children:<br>• Haemoglobin/ anaemia	• Compared with control, positive impact on anaemia prevalence in children (−14.0 percentage points; P=0.02)	This research shows a positive impact of a 22-month EHFP intervention on child's anaemia prevalence and an increase in

Author(s), year, study location	Evaluation design	Intervention	Outcomes measured	Findings	Authors' own conclusions
	homestead food production (EHFP), (2) EHFP + aquaculture, (3) control<br>• Baseline survey and dietary assessment following 22-months of intervention was conducted in 50% of participating women (control: n=140, EHFP n=146, EHFP + aquaculture: n=143) and their children (<5 y).<br>• Haemoglobin and anthropometry measured only in a subset of women and children	and infant and young child feeding.<br>*Agriculture:* agricultural inputs (seeds, seedlings, family tools etc.) and training. EHFP + aquaculture group received training to build fish pond and fish-raising inputs.	• Anthropometry (maternal body mass index, child weight-for-height and weight-for-age)<br>• Non-pregnant women's RBP, serum ferritin, sTfR, AGP, and CRP concentration	and RBP concentrations in women (DID: 0.34; P=0.02) randomized to EHFP and EHFP + aquaculture groups, respectively.<br>• No other impacts on anaemia, nutritional biomarkers, or anthropometry.	RBP concentrations among non-pregnant women receiving EHFP + aquaculture intervention, compared with control.<br>Clinical significance of increases in women's RBP is questionable as no women presented deficiencies at baseline.<br>Longer duration studies are required to explore the full impact of NSA programmes on nutrition status and anthropometry.
Verbowski, et al. 2018, Cambodia[8]	• As Michaux, et al. 2019,[7]	As Michaux, et al. 2019,[7]	Measured in women and children:<br>• Nutrient intake and adequacy of nutrient intake assessed interactive multiple-pass 24-h dietary recall method.	• Compared to controls, women in the EHFP and EHFP + aquaculture had significantly higher zinc and Vitamin A intakes, and women in the EHFP + aquaculture group also had significantly higher iron and riboflavin intakes. No impact on energy (kcal/d), protein, fat, calcium, or thiamin intake.<br>• Women in the EHFP + aquaculture group had significantly lower prevalence of inadequate iron (−7%, at 10% bioavailability), Vitamin A (−19%), and riboflavin (−17%) intakes, compared to controls. No impact on prevalence of inadequate intake for protein, zinc, calcium, and thiamine.<br>• No impact on prevalence of nutrient inadequacy for any nutrients comparing EHFP group to control.	Following 22-month intervention (EHFP and EHFP + aquaculture), increases in certain micronutrient intakes were observed among women, but no effect among children.<br>EHFP + aquaculture group also showed lower prevalence of nutrient inadequacy for certain micronutrients among women.<br>At baseline, very few women showed deficiencies in these micronutrients, thus the overall impact of these observations on health is questionable.

Author(s), year, study location	Evaluation design	Intervention	Outcomes measured	Findings	Authors' own conclusions
				• No impact on nutrient intake or prevalence of nutrient inadequacy among children.	
Rosenberg, et al. 2018, Zambia[9]<br><br>Note this refers to a trial whose primary results were reported previously,[10] covered in Ruel et al. 2018 review.[1]	• Cluster RCT with a control and two treatment groups: (1) agriculture intervention only, (2) integrated agricultural and nutrition interventions<br><br>• Approximately 1,000 households per arm<br><br>• Two cross-sectional surveys four years apart (2011 and 2015)	*Nutrition:* Infant and child young feeding behaviour change communication through women's groups, community health volunteers, and social marketing<br><br>*Agriculture:* Home gardening; inputs included nutrient-rich vegetable, legume, and tuber seeds; tools and training; and goats and chickens and related training<br><br>*Gender:* Promotion of gender equality and women's empowerment	Measured in women and children (6-24 months):<br><br>• Individual dietary diversity score of 7 food groups assessed using 24-hr recall<br><br>Household agricultural production diversity, crop production, sale and consumption from own production, and household food access	• No impact on maternal and child individual dietary diversity score<br><br>• Significant positive effects on, agricultural production diversity, the number of unique food groups grown, consumption of certain crops from own production, and household food access.	A NSA program can increase diversity in agricultural production and to a lesser extent access to nutritious foods, but this may not always be sufficient to improve child diets or nutrition.
Schreinmachers, et al. 2017, Nepal[11]	• Longitudinal cluster RCT<br><br>• 30 schools randomly allocated from eligible list to either (1) phase 1 intervention, (2) phase 2 intervention, (3) control<br><br>• Repeat cross-sectional surveys at start and end of the school year. Baseline sample: Phase 1 intervention n=466, Phase 2 intervention n=400, control n=464<br><br>• Mean child age: 12.5 years and mixed sex	*Nutrition:* Nutrition education and WASH<br><br>Children encouraged to establish a garden at home from the lessons learned in school.<br><br>*Agriculture:* School garden for cultivation of nutrient-dense vegetables. Schools provided with agricultural inputs. Seeds distribution to grow vegetables at home. Education on gardening and agriculture.	Children's:<br><br>• Food knowledge and awareness<br><br>• Fruit and vegetable consumption in previous day measured via 24-h recall<br><br>• Anthropometry (height-for-age)	• Positive impact on children's awareness about nutrition and sustainable agriculture in intervention compared with control.<br><br>• No impact on vegetable consumption or nutritional status.<br><br>• No impact on height-for-age z-scores	School vegetable gardens linked to complementary lessons and promotion are effective as an educational tool but are not enough to improve children's dietary behaviour and nutritional status.

Abbreviations: BCC, behaviour change communication; CBCC, Community-based childcare centres; CF, complementary feeding practices; CM, community mobilisation; CRCT, cluster-randomised controlled trial; DD, dietary diversity; DID, difference in differences; EAR, Estimated average requirements; ECD, early child development; EHFP, enhanced homestead food production; HH, household; IPC, interpersonal communication; ITT, intention to treat; IYCF, infant and young child feeding; LAZ/HAZ,

length-for-age z score/ height-for-age *z*-score; LMIC, low-middle income countries; MDD, minimum dietary diversity; MUACZ, mid-upper arm circumference *z*-score; NSA, nutrition-sensitive agriculture; OR, odds ratio; PA, probability of adequacy; WAZ, weight-for-age *z*-score; WHZ, weight-for-height *z*-score.

[1] Ruel MT, Quisumbing AR, Balagamwala M. Nutrition-sensitive agriculture: What have we learned so far? *Glob Food Sec* 2018; **17**: 128–53.

[2] Gelli A, Nguyen PH, Santacroce M, Twalibu A, Margolies A, Katundu M. A Community-Based Early Childhood Development Center Platform Promoting Diversified Diets and Food Production Increases the Mean Probability of Adequacy of Intake of Preschoolers in Malawi: A Cluster Randomized Trial. *J Nutr* 2019; **150**: 350–5.

[3] Gelli A, Margolies A, Santacroce M, *et al.* Using a Community-Based Early Childhood Development Center as a Platform to Promote Production and Consumption Diversity Increases Children's Dietary Intake and Reduces Stunting in Malawi: A Cluster-Randomized Trial. *J Nutr* 2018; **148**: 1587–97.

[4] Kim SS, Nguyen PH, Yohannes Y, *et al.* Behavior Change Interventions Delivered through Interpersonal Communication, Agricultural Activities, Community Mobilization, and Mass Media Increase Complementary Feeding Practices and Reduce Child Stunting in Ethiopia. *J Nutr* 2019; **149**: 1470–81.

[5] Marquis GS, Colecraft EK, Kanlisi R, *et al.* An agriculture-nutrition intervention improved children's diet and growth in a randomized trial in Ghana. *Matern Child Nutr* 2018; **14**: e12677.

[6] Miller LC, Neupane S, Joshi N, *et al.* Multisectoral community development in Nepal has greater effects on child growth and diet than nutrition education alone. *Public Health Nutr* 2020; **23**: 146–61.

[7] Michaux KD, Hou K, Karakochuk CD, *et al.* Effect of enhanced homestead food production on anaemia among Cambodian women and children: A cluster randomized controlled trial. *Matern Child Nutr* 2019; **15**: e12757.

[8] Verbowski V, Talukder Z, Hou K, *et al.* Effect of enhanced homestead food production and aquaculture on dietary intakes of women and children in rural Cambodia: A cluster randomized controlled trial. *Matern Child Nutr* 2018; **14**. DOI:10.1111/mcn.12581.

[9] Rosenberg AM, Maluccio JA, Harris J, *et al.* Nutrition-sensitive agricultural interventions, agricultural diversity, food access and child dietary diversity: Evidence from rural Zambia. *Food Policy* 2018; **80**: 10–23.

[10] Kumar N, Nguyen PH, Harris J, Harvey D, Rawat R, Ruel MT. What it takes: evidence from a nutrition- and gender-sensitive agriculture intervention in rural Zambia. *J Dev Eff* 2018; **10**: 341–72.

[11] Schreinemachers P, Bhattarai DR, Subedi GD, *et al.* Impact of school gardens in Nepal: a cluster randomised controlled trial. *J Dev Eff* 2017; **9**: 329–43.

Supplementary Table S4 - Trial Indicators

Outcome	Definition
**Primary outcomes**	
Child dietary diversity	Percentage of children (aged 6–23 months) consuming ≥ 4 out of 7 food groups the previous day (assessed by 24-h recall answered by the mother or female primary caregiver). The seven food groups are defined using WHO categories:[1] (1) grains, roots, and tubers; (2) legumes and nuts; (3) dairy products (milk, yoghurt, cheese); (4) flesh foods (meat, fish, poultry, and liver/organ meats); (5) eggs; (6) vitamin-A rich fruits and vegetables; (7) other fruits and vegetables.
Maternal body-mass index	Mean body-mass index (BMI) (kg/m^2) of women aged 15-49 years who are mothers or female primary caregivers of children aged 0–23 months. Excludes women who are pregnant or post-partum (gave birth ≤ 42 days ago).
**Secondary outcomes**	
Maternal dietary diversity	Percentage of mothers or female primary caregivers consuming ≥ 5 out of 10 food groups in the previous 24-hours (measured using a 24-h multi-pass free recall). The ten food groups are defined using the Minimum Dietary Diversity for Women indicator: [2] (1) starchy staples (cereals, white roots and tubers); (2) pulses; (3) nuts and seeds; (4) dairy; (5) flesh foods (meat and fish); (6) eggs; (7) dark green leafy vegetables; (8) other vitamin-A rich fruits and vegetables; (9) other vegetables; (10) other fruits.
Child wasting	Percentage of children (aged 0–23 months) with a weight-for-height z-score < -2 SD)
**Other outcomes**	
Maternal wasting	Percentage of mothers or female primary caregivers with mid-upper arm circumference (MUAC) < 230 mm. [3] Includes pregnant, post-partum, and not pregnant women.
Child acute malnutrition	Percentage of children (aged 6–23 months) with mid-upper arm circumference < 125 mm.[4]
Maternal and child haemoglobin (Hb) concentrations	Mean Hb concentration (g/dl) of children (aged 6–23 months)
	Mean Hb concentration (g/dl) of mothers or female primary caregivers. Excludes pregnant women.
Infant and young child feeding practices	Percentage of children (aged 6–23 months) receiving the World Health Organization-recommended 'Minimum Acceptable Diet' in the previous 24 hours. [1]<br>• If a child is breastfed, their diet is 'acceptable' if they were given: at least four out of seven food groups, and at least two (age 6-8 months) or three (age 9-23 months) 'meals' of solid, semi-solid, or soft foods.<br>• If a child is not breastfed, their diet is 'acceptable' if they were given: at least four out of six food groups (excludes dairy), at least two milk feeds, and at least four 'meals' (including solid, semi-solid, or soft foods and milk feeds).
Women's decision-making	Percentage of women 'empowered' in women's decision-making in productive and health-related domains, defined as at least some input in two or more decisions.

Outcome	Definition
Women's time use	Percentage of women 'empowered' in the women's time use domain of the Women's Empowerment in Agriculture Index, defined as working less than 10.5h in the last 24h.[5]
Gender parity in agriculture	Percentage of women achieving gender parity between themselves and a male household member, measured using select indicators from the Women's Empowerment in Agriculture Index.[6] Parity is achieved when women have equal or higher empowerment scores than men. Empowerment scores are calculated for men and women as weighted sums of five indicators:<br>1. Made at least two decisions about agriculture (weight: 1/4),<br>2. Reported owning at least one large asset or two smaller assets (weight: 2/12),<br>3. Participated in decisions about credit (weight: 1/12),<br>4. Active member of at least one group (weight: 1/4)<br>5. Worked less than 10.5h in the previous 24h (weight: 1/4).
Household food security and economic status	Mean share of total household expenditures spent on food (proportion)
	Mean per capita daily household expenditures on food and non-food items (INR)
Household agriculture production	Mean production diversity score, calculated as the count of the number of crops or livestock produced out of 10 food groups from the Minimum Dietary Diversity Score for Women.[2]
	Total value of agricultural production in the last three agricultural seasons, calculated as the sum of quantity × price for each item produced (INR).
	Net value of agricultural production in the last three agricultural seasons, calculated as value of agricultural production minus costs of agricultural inputs (INR).

[1] World Health Organization. Indicators for assessing infant and young child feeding practices Part 2: Measurement. World Health Organization, Geneva. 2010.

[2] FAO and FHI 360. Minimum Dietary Diversity for Women: A Guide to Measurement. Rome: FAO. 2016 www.fao.org/publications (accessed May 27, 2020).

[3] Tang AM, Dong K, Deitchler M, *et al.* Use of Cutoffs for Mid-Upper Arm Circumference (MUAC) as an Indicator or Predictor of Nutritional and Health-Related Outcomes in Adolescents and Adults: A Systematic Review. Washington, DC: F. 360/FANTA; 2013. www.fantaproject.org (accessed May 27, 2020).

[4] WHO and UNICEF. WHO child growth standards and the identification of severe acute malnutrition in infants and children: WHO child growth standards and the identification of severe acute malnutrition in infants and children: joint statement by the World Health Organization and the United Nations Children's Fund. 2009 www.who.int/childgrowth/standards (accessed May 27, 2020).

[5] Malapit H, Kovarik C, Sproule K, Meinzen-Dick R, Quisumbing A. Instructional Guide on the Abbreviated Women's Empowerment in Agriculture Index (A-WEAI) 1. 2015 http://www.ifpri.org/publication/measuring-progress-toward-empowerment (accessed May 28, 2020).

[6] Malapit HJ, Kovarik C, Sproule K, Meinzen-Dick RS, Quisumbing AR. Instructional Guide on the Abbreviated Women's Empowerment in Agriculture Index (A-WEAI). Washington, DC: International Food Policy Research Institute; 2015. https://www.ifpri.org/publication/instructional-guide-abbreviated-womens-empowerment-agriculture-index-weai (accessed May 27, 2020).

Supplementary Table S5 - Demographic characteristics of households at endline

Characteristics	All arms N=4291	Control N=997	AGRI N=1100	AGRI-NUT N=1,055	AGRI-NUT+PLA N=1139
**Proportion of Scheduled Caste or Scheduled Tribe households in the village at baseline, n (%)**					
Low (<30%)	563 (13%)	138 (14%)	104 (9%)	170 (16%)	151 (13%)
Medium (30-70%)	2487 (58%)	626 (63%)	606 (55%)	585 (55%)	670 (59%)
High (>70%)	1241 (29%)	233 (23%)	390 (35%)	300 (28%)	318 (28%)
**Distance of village to nearest town at baseline, n (%)**					
<10 km	814 (19%)	180 (18%)	190 (17%)	180 (17%)	264 (23%)
≥10 km	3477 (81%)	817 (82%)	910 (83%)	875 (83%)	875 (77%)
**Block, n (%)**					
Ghatagaon	1536 (36%)	270 (27%)	387 (35%)	442 (42%)	437 (38%)
Harichandanpur	1693 (39%)	431 (43%)	401 (36%)	382 (36%)	479 (42%)
Patna	608 (14%)	199 (20%)	145 (13%)	81 (8%)	183 (16%)
Keonjhar (Sardar)	454 (11%)	97 (10%)	167 (15%)	150 (14%)	40 (4%)
**Child's sex, n (%)**					
Male	2176 (51%)	498 (50%)	553 (50%)	548 (52%)	577 (51%)
Female	2115 (49%)	499 (50%)	547 (50%)	507 (48%)	562 (49%)
**Child's age**					
Completed months, mean (SD)	12·0 (6·7)	12·1 (6·7)	11·7 (6·7)	12·2 (6·8)	12·0 (6·8)
**Mother's age**					
Completed years, mean (SD)	24·7 (4·3)	24·9 (4·1)	24·7 (3·4)	24·5 (4·1)	24·8 (4·4)
**Education of mother**					
Completed years, mean (SD)	6·9 (4·5)	7·1 (4·5)	6·6 (4·5)	7·0 (4·3)	6·9 (4·6)
**Education of spouse**					
Completed years, mean (SD)	7·5 (4·0)	7·8 (4·0)	7·3 (4·2)	7·4 (3·9)	7·6 (4·0)
Missing	4 (0%)	1 (0%)	2 (0%)	0 (0%)	1 (0%)
**Household land ownership**					
Owns any land, n (%)					
No	11 (0%)	5 (1%)	2 (0%)	2 (0%)	2 (0%)
Yes	4280 (100%)	992 (99%)	1098 (100%)	1053 (100%)	1137 (100%)
Acres of land owned (if any), median (IQR)	1·08 (0·50, 2·05)	1·05 (0·44, 2·00)	1·06 (0·50, 2·05)	1·15 (0·56, 2·06)	1·06 (0·45, 2·10)
>0 - < 2·50 acres, n (%)	3440 (80%)	816 (82%)	878 (80%)	842 (80·0%)	904 (79%)
2·50 - 5·00 acres, n (%)	618 (14%)	141 (14%)	154 (14%)	149 (14·1%)	174 (15%)
> 5·00 acres, n (%)	218 (5%)	34 (3%)	66 (6%)	60 (5·7%)	58 (5%)
Acres of land owned unknown	4 (0%)	1 (0%)	0 (0%)	2 (0·2%)	1 (0%)
**Asset ownership**					
Sum of 17 assets, mean (SD)	9·7 (2·6)	9·5 (2·7)	9·7 (2·6)	9·8 (2·5)	9·8 (2·6)
Missing	4 (0%)	1 (0%)	0 (0%)	2 (0%)	1 (0%)
**Household size**					
Total number of household members, mean (SD)	5·2 (1·7)	5·1 (1·6)	5·3 (1·8)	5·1 (1·7)	5·1 (1·7)
**Household composition, n (%)**					

Characteristics	All arms<br>N=4291	Control<br>N=997	AGRI<br>N=1100	AGRI-NUT<br>N=1,055	AGRI-NUT+PLA<br>N=1139
Contains male and female adults	4040 (94%)	944 (95%)	1043 (95%)	998 (95%)	1055 (93%)
Contains female only adults	247 (6%)	52 (5%)	57 (5%)	55 (5%)	83 (7%)
Missing	4 (0%)	1 (0%)	0 (0%)	2 (0%)	1 (0%)

Data are n (%), mean (SD), or median (IQR).

Supplementary Table S6 – Changes between baseline and endline in the primary, secondary, and other outcomes across all trial arms adjusted for the stratification factors (caste and distance from nearest town), after accounting for any differences attributable to the interventions

	**Endline vs baseline**	
	**Effect (95% CI)**	**p value**
**Primary Outcomes**		
Child minimum dietary diversity (ate ≥4 food groups) *	1·33 (1·18, 1·50)	<0·0001
Maternal body-mass index †	0·25 (0·04, 0·45)	0·017
**Secondary outcomes**		
Maternal minimum dietary diversity (ate ≥5 food groups)	1·50 (1·29, 1·74)	<0·0001
Wasting (weight-for-height z-score <-2 SD)	0·92 (0·74, 1·13)	0·43
**Other outcomes**		
Maternal low MUAC, <230 mm	0·99 (0·91, 1·06)	0·73
Child low MUAC, <125 mm *	1·49 (1·15, 1·94)	0·00026
Maternal haemoglobin (g/dl) ‡	0·04 (-0·07, 0·14)	0·53
Child haemoglobin (g/dl) *	-0·23 (-0·37, -0·10)	0·00065
Child given minimum acceptable diet *	1·72 (1·49, 1·99)	<0·0001
Women made ≥ 2 decisions in agriculture or health	1·01 (0·97, 1·05)	0·77
Women worked <10·5h in the previous 24h	0·94 (0·79, 1·12)	0·49
Women achieving gender parity in agriculture §	1·39 (1·26, 1·53)	<0·0001
Share of household expenditures spent on food ¶	0·03 (0·01, 0·05)	0·018
Per capita total daily household expenditure (INR) ¶ ||	-1·15 (-3·22, 0·93)	0·27
Agricultural production diversity out of 10 food groups over 1 year	-0·40 (-0·67, -0·14)	0·0032
Total value of agricultural production over 1 year (INR) ||	11393 (2191, 16609)	0·0014
Net value (total value minus input costs) of agriculture production over 1 year (INR) ||	8362 (-606, 13344)	0·017

Data are mean (SD), n/N (%), or median (IQR). MUAC=mid-upper arm circumference. INR=Indian Rupees. All denominators include responses from the endline survey and with valid measurements for each outcome. * Includes children 6-23 months only. † Includes non-pregnant, non-postpartum women only. ‡ Includes non-pregnant women only. § Gender parity achieved when women have equal or higher empowerment scores than men. Empowerment scores are calculated for men and women as weighted sums of five indicators: decision making, asset ownership, access to credit, group membership, and time use (empowerment questionnaire only excluding records with female respondent to male questionnaire). Measured in 50% of households; excludes female-only households. ¶ Measured in 50% of households. || Confidence intervals are bias corrected estimated using non-parametric bootstrapping.

Supplementary Table S7 - Effect of interventions only adjusted for baseline measures of the outcome

	Control	AGRI	AGRI-NUT	AGRI-NUT+PLA	AGRI vs control		AGRI-NUT vs control		AGRI-NUT+PLA vs control	
					Effect (95% CI)	p value	Effect (95% CI)	p value	Effect (95% CI)	p value
**Primary Outcomes**										
Child minimum dietary diversity (ate ≥4 food groups) *	286/757 (38%)	325/822 (40%)	359/812 (44%)	413/863 (48%)	1·06 (0·91, 1·23)	0·48	1·19 (1·03, 1·37)	0·02	1·26 (1·10, 1·45)	0·001
Maternal body-mass index †	19·5 (2·81) n=923	19·3 (2·90) n=1014	19·4 (2·99) n=978	19·4 (2·92) n=1035	-0·06 (-0·36, 0·24)	0·68	0·04 (-0·26, 0·33)	0·81	-0·05 (-0·31, 0·22)	0·74
**Secondary outcomes**										
Maternal minimum dietary diversity (ate ≥5 food groups)	331/997 (33%)	396/1100 (36%)	402/1055 (38%)	479/1139 (42%)	1·20 (0·99, 1·45)	0·059	1·17 (0·98, 1·39)	0·084	1·29 (1·09, 1·53)	0·0025
Wasting (weight-for-height z-score <-2 SD)	133/986 (13%)	152/1096 (14%)	138/1052 (13%)	158/1133 (14%)	0·95 (0·73, 1·24)	0·71	0·96 (0·71, 1·29)	0·79	0·95 (0·72, 1·25)	0·72
**Other outcomes**										
Maternal low MUAC, <230 mm	445/989 (45%)	539/1096 (49%)	465/1051 (44%)	525/1134 (46%)	1·08 (0·96, 1·23)	0·21	0·96 (0·85, 1·08)	0·48	1·01 (0·91, 1·13)	0·84
Child low MUAC, <125 mm *	96/783 (12%)	128/861 (15%)	104/827 (13%)	110/893 (12%)	1·17 (0·86, 1·59)	0·31	1 (0·76, 1·31)	0·99	0·93 (0·66, 1·30)	0·65
Maternal haemoglobin (g/dl) ‡	11·7 (1·30) n=974	11·6 (1·29) n=1077	11·6 (1·30) n=1033	11·6 (1·29) n=1111	0·01 (-0·16, 0·18)	0·88	0 (-0·17, 0·16)	0·98	-0·13 (-0·30, 0·04)	0·14
Child haemoglobin (g/dl) *	10·1 (1·19) n=776	10·2 (1·29) n=857	10·1 (1·26) n=825	10·1 (1·25) n=883	0·10 (-0·07, 0·28)	0·24	0·04 (-0·14, 0·21)	0·68	-0·05 (-0·24, 0·14)	0·60
Child given minimum acceptable diet *	268/790 (40%)	290/862 (34%)	327/829 (39%)	384/895 (43%)	1·01 (0·85, 1·20)	0·90	1·20 (1·01, 1·42)	0·035	1·29 (1·10, 1·52)	0·0017
Women made ≥ 2 decisions in agriculture or health	867/997 (87%)	1015/1100 (92%)	961/1055 (91%)	1002/1139 (88%)	1·05 (1·00, 1·11)	0·044	1·05 (0·99, 1·11)	0·11	1·02 (0·97, 1·07)	0·53

	Control	AGRI	AGRI-NUT	AGRI-NUT+PLA	AGRI vs control		AGRI-NUT vs control		AGRI-NUT+PLA vs control	
Women worked <10·5h in the previous 24h	339/997 (34%)	339/1100 (31%)	332/1055 (31%)	357/1139 (31%)	0·79 (0·60, 1·05)	0·10	0·85 (0·62, 1·16)	0·30	0·76 (0·56, 1·03)	0·079
Women achieving gender parity in agriculture §	253/406 (62%)	283/439 (64%)	264/419 (63%)	294/465 (63%)	1·02 (0·92, 1·13)	0·72	1·00 (0·89, 1·13)	0·95	0·98 (0·88, 1·10)	0·71
Share of household expenditures spent on food ¶	0·62 (0·17) n=500	0·63 (0·17) n=546	0·62 (0·17) n=531	0·63 (0·17) n=569	0·00 (-0·04, 0·03)	0·83	-0·01 (-0·04, 0·03)	0·68	0·01 (-0·02, 0·04)	0·65
Per capita total daily household expenditure (INR) ¶ ‖	17·6 (12·9, 25·7) n=500	18·2 (13·5, 26·6) n=546	18·2 (13·4, 26·7) n=531	18·2 (12·6, 26·1) n=569	0·96 (-1·85, 3·49)	0·48	0·85 (-1·59, 3·25)	0·50	2·38 (-0·42, 5·40)	0·10
Agricultural production diversity out of 10 food groups over 1 year	4·1 (2·03) n=996	4·4 (2·03) n=1100	4·6 (1·95) n=1053	4·4 (2·02) n=1138	0·13 (-0·25, 0·51)	0·50	0·37 (-0·01, 0·75)	0·054	0·31 (-0·07, 0·70)	0·11
Total value of agricultural production over 1 year (INR) ‖	16696 (6312, 34400) n=996	19465 (8475, 37648) n=1100	19668 (9229, 38368) n=1053	17378 (7395, 34683) n=1138	7472 (2154, 16233)	0·032	-344 (-9453, 8180)	0·94	4898 (-230, 13378)	0·15
Net value (total value minus input costs) of agriculture production over 1 year (INR) ‖	10048 (2810, 24241) n=996	13489 (4405, 28118) n=1100	13581 (4792, 27869) n=1053	11885 (3528, 25779) n=1138	6755 (1742, 15348)	0·047	-221 (-9214, 7854)	0·96	5323 (801, 13850)	0·11

Data are mean (SD), n/N (%), or median (IQR). MUAC=mid-upper arm circumference. INR=Indian Rupees. All denominators include responses from the endline survey and with valid measurements for each outcome. * Includes children 6-23 months only. † Includes non-pregnant, non-postpartum women only. ‡ Includes non-pregnant women only. § Gender parity achieved when women have equal or higher empowerment scores than men. Empowerment scores are calculated for men and women as weighted sums of five indicators: decision making, asset ownership, access to credit, group membership, and time use (empowerment questionnaire only excluding records with female respondent to male questionnaire). Measured in 50% of households; excludes female-only households. ¶ Measured in 50% of households. ‖ Confidence intervals are bias corrected estimated using non-parametric bootstrapping.

Supplementary Table S8 – Per protocol analysis adjusted for baseline measures of the outcomes and stratification factors (caste and distance from nearest town)

		**Child minimum dietary diversity ***			**Maternal minimum dietary diversity**		
		n/N (%)	Effect	p value	n/N (%)	Effect	p value
**Control**		286/757 (38%)	1	NA	331/997 (33%)	1	NA
**AGRI**	no exposure	147/396 (37%)	0·99 (0·81, 1·22)	0·96	168/554 (30%)	1·00 (0·79, 1·26)	0·98
	exposure to videos	178/426 (42%)	1·13 (0·95, 1·34)	0·17	228/546 (42%)	1·30 (1·07, 1·58)	0·009
**AGRI-NUT**	no exposure	130/342 (38%)	1·02 (0·84, 1·24)	0·83	149/448 (33%)	1·02 (0·80, 1·29)	0·89
	exposure to videos	229/470 (49%)	1·29 (1·11, 1·51)	0·001	253/607 (42%)	1·30 (1·08, 1·57)	0·005
**AGRI-NUT+PLA**	no exposure	173/389 (44%)	1·21 (1·02, 1·44)	0·026	186/507 (37%)	1·15 (0·91, 1·44)	0·24
	exposure to videos only	157/310 (51%)	1·36 (1·15, 1·60)	<0·0001	197/419 (47%)	1·44 (1·17, 1·76)	<0·0001
	exposure to PLA only	3/5 (60%)	1·58 (0·86, 2·93)	0·14	2/5 (40%)	1·27 (0·37, 4·33)	0·70
	exposure to PLA and videos	80/159 (50%)	1·36 (1·08, 1·70)	<0·0001	94/208 (45%)	1·48 (1·19, 1·85)	<0·0001

Data are n/N (%). PLA=participatory learning and action. * Includes children 6-23 months only.

Supplementary Table S9a - Components of total costs by intervention arm

Cost Description	Total costs An annual discount rate of 3%		AGRI		AGRI+NUT		AGRI+NUT+PLA	
			Total costs		Total costs		Total costs	
	2019 INT$	%	2019 INT$	%	2019 INT$	%	2019 INT$	%
**Costs by Implementation phase**								
**Start-up**	497,837	21%	130,739	20%	187,415	24%	179,683	18%
**Implementation**	1,917,812	79%	508,802	80%	601,828	76%	807,182	82%
**Costs by Line item**								
**Staff**	1,584,464	66%	420,790	66%	489,431	62%	674,243	68%
**Materials**	23,975	1%	7,675	1%	7,670	1%	8,630	1%
**Capital**	168,363	7%	35,903	6%	54,800	7%	77,660	8%
**Other recurrent**	638,847	26%	175,172	27%	237,342	30%	226,333	23%

Supplementary Table S9b - Cost description and unit costs of the UPAVAN interventions (2019 INT$)

Cost Description	AGRI	AGRI+NUT	AGRI+NUT+PLA
**Total cost**	639,541	789,243	986,865
**Total cost per person covered (all ages)**	16	20	21
**Total cost per pregnant women and mothers of children under 2 years of age**	146	182	199
**Annual total costs, averaged over 41 months of the study period**	187,182	230,998	288,838
**Annual implementation costs, averaged over 32 months of the intervention period**	190,801	225,685	302,693
**Annual cost per person covered (all ages)**	5	6	6
**Annual cost per pregnant woman and mother of children under 2 years of age**	43	53	58
